# Regulation of cellular senescence by eukaryotic members of the FAH superfamily – A role in calcium homeostasis?

**DOI:** 10.1016/j.mad.2020.111284

**Published:** 2020-06-20

**Authors:** Alexander K.H. Weiss, Eva Albertini, Max Holzknecht, Elia Cappuccio, Ilaria Dorigatti, Anna Krahbichler, Elisabeth Damisch, Hubert Gstach, Pidder Jansen-Dürr

**Affiliations:** a University of Innsbruck, Research Institute for Biomedical Aging Research, Rennweg 10, A-6020, Innsbruck, Austria; b University of Innsbruck, Center for Molecular Biosciences Innsbruck (CMBI), Austria; c University of Vienna, UZ2 E349, Department of Pharmaceutical Chemistry, Faculty of Life Sciences, Althanstrasse 14, 1090, Vienna, Austria

**Keywords:** FAHD1, FAHD2, FAH superfamily, Calcium homeostasis

## Abstract

*Fumarylacetoacetate hydrolase* (FAH) superfamily members are commonly expressed in the prokaryotic kingdom, where they take part in the committing steps of degradation pathways of complex carbon sources. Besides FAH itself, the only described FAH superfamily members in the eukaryotic kingdom are *fumarylacetoacetate hydrolase domain containing proteins* (FAHD) 1 and 2, that have been a focus of recent work in aging research. Here, we provide a review of current knowledge on FAHD proteins. Of those, FAHD1 has recently been described as a regulator of mitochondrial function and senescence, in the context of mitochondrial dysfunction associated senescence (MiDAS). This work further describes data based on bioinformatics analysis, 3D structure comparison and sequence alignment, that suggests a putative role of FAHD proteins as calcium binding proteins.

## 1 Introduction

### 1.1 Identification of FAHD1 as regulator of mitochondrial function

In 1959 and 1974, Corwin and Wojtczak identified a mitochondrial oxaloacetate decarboxylase from rat liver (Corwin, 1959; Anna and Wojtczak, 1974). This was about 60 years ago, and until recently the identity of the enzyme remained unclear. In 2007 high resolution 2D gels of mitochondrial preparations from young and senescent human umbilical vein endothelial cells (HUVEC) were prepared using the *ProteoTope* ™ technique (Groebe et al., 2007). This revealed an age-related difference in isoelectric point of about 0.4 pI units for two protein spots (#1756 and #1780/1784) (Groebe et al., 2007; Etemad et al., 2019), suggesting differences in post-translational modification of the associated protein with cellular senescence. Mass spectrometric analysis identified the protein as *fumarylacetoacetate hydrolase domain containing protein 1* (FAHD1) (Pircher et al., 2011). In 2011 and 2015, Pircher et al. were able to identify FAHD1 as acylpyruvate hydrolase (ApH) and oxaloacetate decarboxylase (ODx), which is localized in mitochondria (Pircher et al., 2011) and belongs to the broad FAH superfamily of enzymes (Pircher et al., 2011; Kang et al., 2011; Hong et al., 2020; Pircher et al., 2015; Timm et al., 1999; Bateman et al., 2001). The localization of FAHD1 in mitochondria (Pircher et al., 2011) and its ODx activity rendered a model of FAHD1 acting as regulator of oxaloacetate levels in the TCA cycle (Etemad et al., 2019; Pircher et al., 2015; Jansen-Duerr et al., 2016), which was accompanied by the description of the FAHD1 catalytic mechanism (Weiss et al., 2018a). Work with the model organism *Caenorhabditis elegans* provided first support for this hypothesis, as deletion of *fahd-1* induced severe mitochondrial dysfunction and impaired locomotion activity (Taferner et al., 2015). Recent work linked FAHD-1 activity to serotonin signaling in the nematode (Baraldo et al., 2019). Work with human endothelial cells (HUVEC) displayed that depletion of FAHD1 inhibits mitochondrial electron transport chain (ETC) and induces cellular senescence in human endothelial cells (Petit et al., 2017). This enabled the hypothesis of FAHD1 being a regulator of cellular senescence *via* regulation of the mitochondrial ETC (Etemad et al., 2019) in the context of mitochondrial dysfunction associated senescence (MiDAS) described previously by us (Stöckl et al., 2006) and others (Wiley et al., 2016).

Oxaloacetate decarboxylases are mainly known from prokaryotic organisms, where membrane-bound (Lietzan and St Maurice, 2014) and soluble variants exist (Klaffl and Eikmanns, 2010). The membrane-bound variants generally depend on sodium ions and biotin, whereas the soluble variants depend on bivalent metal cations (Weiss et al., 2018b) such as Mg^2+^, Ca^2+^, and Mn^2+^. The described eukaryotic members of the FAH superfamily are FAH, FAHD1, and FAHD2. FAHD1 differs from FAH in its physical properties, localization, and rather low catalytic activity (Weiss et al., 2019), which will be discussed in this article. The bi-functionality of FAHD1, acting as ApH and ODx (Weiss et al., 2018a), even raised the idea of the eukaryotic FAHD1 being a hybrid of related prokaryotic precursor proteins (Weiss et al., 2018b). Recent work by Hong et al. (Hong et al., 2020) supports this idea *via* a phylogenetic tree analysis of FAH superfamily enzymes.

However, the exact role of FAHD proteins, and of FAHD1 in particular, is not fully revealed to date. Here, we provide a review of collected data on FAHD proteins in eukaryotes, describing FAHD1 as a regulator of the TCA cycle flux in the context of mitochondrial dysfunction associated senescence. We further present conclusive data obtained *via* bioinformatic analyses, in order to hypothesize a secondary role of FAHD1 as possible calcium binding protein. Published links between calcium metabolism, mitochondrial dysfunction, and cellular senescence are highlighted. This model will extend the role of FAHD1 as a putative regulator of the TCA cycle flux by suggesting multiple physiological functions of FAHD proteins in eukaryotes.

### 1.2 FAHD1 catalytic mechanism revealed by structural studies and site directed mutagenesis

FAHD1 acts bi-functional as ApH and ODx (Weiss et al., 2018a). While ApH activity is common for the FAH superfamily of enzymes in prokaryotes (Hong et al., 2020), ODx activity is not common in the prokaryotic part of the family (except for individual members such as Cg1458 (Ran et al., 2013, 2011) in *Corynebacterium glutamicum*). ODx activity is now well understood in the eukaryotic members of the superfamily (Weiss et al., 2018b), in particular for FAHD1, while the role of ApH activity in the metabolism of eukaryotes remains elusive.

The postulated mechanism for FAHD1 catalytic activity (Weiss et al., 2018a) was substantiated by experimental data. Mutations of particular amino acids by replacement with alanine create enzymatic forms with strongly decreased ODx activity, which are often inactive for the hydrolysis of acylpyruvates (Weiss et al., 2018a). In all enzymes of the FAH superfamily of proteins, highly conserved carboxylate side chains are provided for binding of divalent cations (*e.g.* Mg^2+^, Ca^2+^, Mn^2+^, Zn^2+^, Cu^2+^) (Hong et al., 2020; Weiss et al., 2018b). However, for execution of the specific catalytic functions FAH superfamily members prefer distinct metals. For FAH, Ca^2+^ and Mg^2+^ are functional metal ions. FAHD1 shows highest catalytic activity with Mg^2+^ and Mn^2+^ as cofactors (Pircher et al., 2011). The metal cofactor (Mg^2+^) is held in place by the side chains of the three amino acids E71, E73 and D102 (Weiss et al., 2018a). The substrates of FAHD1, oxaloacetate (OAA) as well as acylpyruvates (Ap), adopt different forms in varying ratio depending on the prevailing pH-value. Under mitochondrial pH of about 7.8 Ap and OAA are competent to bind tightly in divalent binding mode to the cofactor Mg^2+^ of FAHD1. Upon this primary binding event of the substrate, FAHD1 acquires catalytic competence through backbone-flip induced lid closure (Weiss et al., 2018a). This event structures the disordered region of the apo-enzyme and isolates the catalytic cavity from the mitochondrial environment. Structuring of the disordered region induces a short helical region (Weiss et al., 2018a). Helix residues E33 and H30 form a well-known catalytically competent acid-base dyad which interacts through hydrogen bonding with an isolated water molecule in the catalytic center (Weiss et al., 2018a). To prepare for the break of the C^3^–C^4^ bond, the enzyme has to provide a conformational control over the bound substrates *via* Q109. The corresponding mutation Q154A in Cg1458 (Ran et al., 2013) abolished ODx activity. R106 forms hydrogen bonds with E73 and Q109, which is a key feature for maintaining the tertiary structure of the binding pocket (Weiss et al., 2018a). K123 plays a significant role as proton source in the FAHD1 catalytic mechanism. Accordingly, substitution of K123 by alanine creates inactive forms both for ApH and ODx activities (Weiss et al., 2018a).

Deliberate modulation of FAHD1 catalytic activity by selective single-point mutation helps to further understand the role of FAHD1 in mitochondria and prepares for future work with *in vivo* models. Comparing the activity of FAHD1 mutations with respect to the wild type in nematode and mouse will provide evidence for the postulated downstream effects. In parallel, current attempts to develop small molecules with the ability to increase or decrease FAHD1 catalytic activity aim at translational strategies to fine tune FAHD1 activity in particular physiological and pathological conditions.

## 2 FAHD1 and FAHD2: unequal members of the eukaryotic FAH superfamily

### 2.1 FAHD1 and FAHD2 proteins share the FAH fold

Homology search and sequence analysis of FAHD1 with proteins encoded in the genome of mammals revealed a high level of 97 % sequence identity with a putatively cytosolic enzyme: FAH domain containing protein 2 (FAHD2), which is expressed in the human genome in two unrelated versions (a, and b). Both hFAHD2a and hFAHD2b are encoded on human chromosome 2 (GRCh38:CM000664.2) (Uhlen et al., 2015, 2005). hFAHD2a is transcribed in direct sense (95,402,721−95,416,616) and hFAHD2b in reverse (97,083,583−97,094,882).

**Fig. 1:**
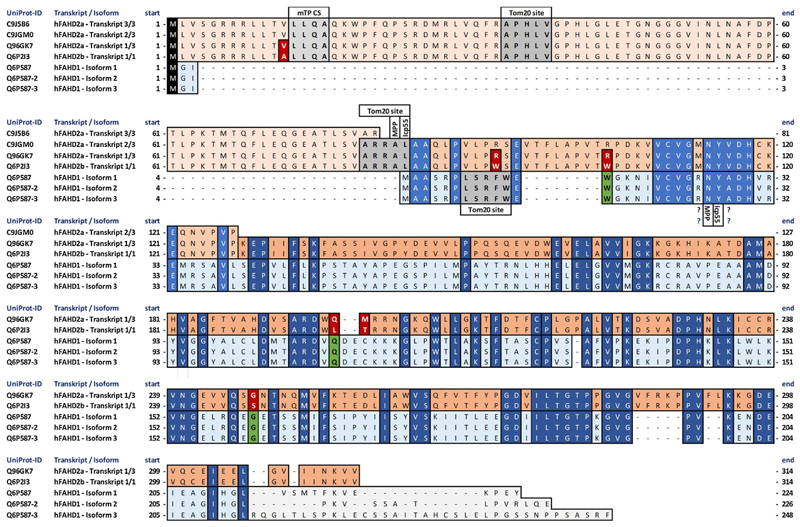
Multiple sequence alignment of human FAHD2a, FAHD2b and FAHD1 isoforms. Human FAHD2 is expressed in two very similar, yet independent forms: FAHD2a and FAHD2b. Three active transcripts can be found for FAHD2a, and one for FAHD2b. Human FAHD1 is expressed in three isoforms. FAHD2a seems to be a hybrid form, consisting of a highly hydrophobic *N*-terminal sequence of 80 amino acids, fused to the actual FAHD protein. Transcripts 2 and 3 of FAHD2a translate to only the hydrophobic part, for which only transcript 1 of FAHD2a and FAHD2b translate to real FAHD proteins (see text). FAHD2a transcript 1 and FAHD2b differ in 6 amino acids marked with red boxes. FAHD proteins display TOM20 sites, which have been found *via* bioinformatics comparison of amino acid sequences (Holzknecht et al., 2018; Dorigatti et al., 2018), as well as sites for proteolytic cleavage of the targeting signal, performed by *mitochondrial processing peptidase* (MPP) and for cleavage of destabilizing *N*-terminal amino acid residues by *intermediate cleaving peptidase 55* (ICP55), which is critical for stabilization of the mitochondrial proteome (Wasmuth and Lima, 2017) (see also Table 1). However, a possible cleavage of FAHD1 by MPP at amino acids N26 and Y27 would destroy the catalytic domain that is required for a functional protein (Weiss et al., 2018a), which appears unlikely. Cleavage of FAHD2 proteins by MPP and ICP55 is plausible, as also the *TargetP-2.0* (Almagro Armenteros et al., 2019) server predicts the presence of a conserved mitochondrial transit peptide sequence (mTP CS) (see panel C of Figure S3) around L14 of FAHD2a and FAHD2b, but not in the sequence of FAHD1.

We found 4 active transcripts for hFAHD2a and 2 active transcripts for hFAHD2b. In both cases two of the active transcripts encode the same protein information, which leads to three forms of hFAHD2a (Q96GK7, C9JGM0 and C9J5B6) and only one form of hFAHD2b (Q6P2I3) (Uhlen et al., 2015, 2005). Transcripts 2 and 3 of hFAHD2a (C9JGM0 and C9J5B6) do not include the FAH fold (see Fig. 1), so only transcript 1 of hFAHD2a (Q96GK7) and the one transcript of hFAHD2b (Q6P2I3) display homology with hFAHD1 (Q6P587). We conclude that both FAHD2a and FAHD2b are homologs to FAHD1. Of interest, sequence comparison of transcript 1 of hFAHD2a with hFAHD2b reveals a difference in only 6 amino acids. The question of why the human genome encodes two such similar proteins on different parts of the same chromosome remains elusive.

The protein structure of FAHD2a and FAHD2b is yet unreported, however, *Swiss-Model* (Waterhouse et al., 2018) homology modelling of the protein structure of FAHD2a (transcript 1, Q96GK7) reveals a strong structural similarity with FAHD1 (see panels A and B of Figure S3). All critical amino acids and structure motifs, that have been identified to be of importance for the catalytic activity of FAHD1, are fully conserved (see Fig. 1). As a result of similarities with FAHD1, Mg^2+^ and Mn^2+^ have been inferred as cofactors, and present data allows for the hypothesis of a similar enzymatic activity. Human FAHD2 manifests an *N-* terminal part, which is not present in human FAHD1 and which probably confers to the protein a strong hydrophobic character (see Fig. 1). In fact, this protein fragment also comprises TOM20 sites, which have been found *via* bioinformatics comparison of amino acid sequences (Holzknecht et al., 2018; Dorigatti et al., 2018) (see Table 1 and section 2.3). The *TargetP-2.0* (Almagro Armenteros et al., 2019) server predicts the presence of a mitochondrial transit peptide (mTP) (see panel C of figure S3) around L14 of FAHD2a and FAHD2b, but not in the sequence of FAHD1.

Human FAHD2a was found to be highly expressed in tissue of liver, testicles and thyroid (Uhlen et al., 2015, 2005), and seems to be overexpressed in cancer tissue compared to benign tissue in different types of cancer such as colorectal, breast, prostate, lung and liver cancer (Uhlen et al., 2015, 2005). Subcellular localization of FAHD2a and FAHD2b has yet to be investigated. While we have collected important information on FAHD1 structure and activity, FAHD2 is highly understudied. Scarce data is available for its catalytic activity, subcellular localization and expression (Fagerberg et al., 2014). A detailed functional characterization of FAHD2a will be required to increase our understanding of the overall role of FAHD proteins.

A survey of mitochondrial TCA cycle enzymes is given in Table 2, comparing the reported structure and predicted stability in solution at physiological conditions. Structure and general protein information has been obtained from the *UniProt* (Wasmuth and Lima, 2017) database. Theoretical pI and stability predictions have been computed using the *ProtParam* (Gasteiger et al., 2005) server. FAHD proteins are predicted to be unstable (Table 2, marked in red), however, FAHD1 is understood to form a soluble and catalytically active homodimer (Pircher et al., 2011, 2015; Weiss et al., 2018a; Manjasetty et al., 2004), whereas all other unstable proteins are part of larger protein complexes (Wasmuth and Lima, 2017) (Table 2, marked in green).

### 2.2 Subcellular localization of FAHD proteins: mitochondria and more?

**Fig. 2:**
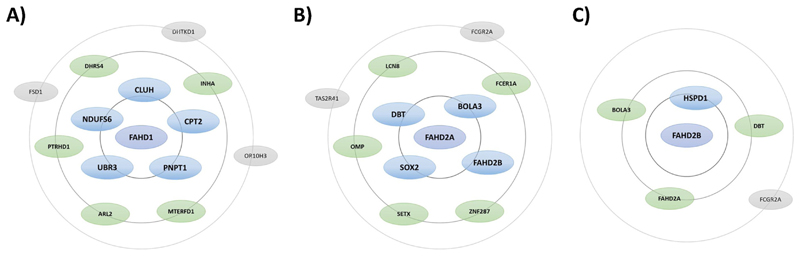
Predicted interaction partners of FAHD proteins. The most probable interaction partners of FAHD proteins according to data analysis by the *BioPlex* (Huttlin et al., 2015) network, are depicted as a bubble chart diagram. Certain proteins have been listed in previous versions of the *BioPlex* (Huttlin et al., 2015) network, but have been removed in newer versions. Taking these changes into account, the probability of interaction partners may be ranked, preferring proteins that are listed in newer versions over proteins that were dropped in newer versions. In each panel, outer circles represent a lower ranking compared with the inner circles.

Subcellular localization of FAHD1 was assessed *via* immunofluorescence by the *Human Protein Atlas* (Uhlen et al., 2005; Fagerberg et al., 2014; Uhlen et al., 2010). Using antibodies HPA043534 and CAB025530, FAHD1 was described to be localized primarily in mitochondria with a potential secondary localization in the nucleoplasm. The localization of potential interaction partners of FAHD1, as listed in the *BioPlex* (Huttlin et al., 2015) network (Table 3, Fig. 2; see also below), generally matches the data reported for FAHD1 subcellular localization, *i.e.*, mitochondria and nucleoplasm; moreover, this annotation is also supported by information about localization and function of the interacting proteins, as gathered from the *Human Protein Atlas* (Uhlen et al., 2005; Fagerberg et al., 2014; Uhlen et al., 2010) and the *UniProt* (Wasmuth and Lima, 2017) database.

A survey of predicted mitochondrial targeting sequences and their cleavage sites using the *MitoFates* (Fukasawa et al., 2015) server is given in Table 1. FAHD proteins display TOM20 binding sites, which have been found *via* bioinformatics comparison of amino acid sequences (Holzknecht et al., 2018; Dorigatti et al., 2018) (see Table 1). TOM20 subunits form a hydrophobic binding pocket in the outer mitochondrial membrane and are central components of the TOM receptor complex (Seki et al., 1995), that is responsible for the recognition and translocation of mitochondrial pre-proteins synthesized in the cytosol or close to the outer mitochondrial membrane (Lesnik et al., 2015) (see section 2.1).

Both FAHD1 and FAHD2 display sites for proteolytic cleavage of the targeting signal, performed by *mitochondrial processing peptidase* (MPP), as well as sites for cleavage of destabilizing *N*-terminal amino acid residues by *intermediate cleaving peptidase 55* (ICP55), which is critical for stabilization of the mitochondrial proteome (Wasmuth and Lima, 2017) (see also Fig. 1). Interestingly, while both the FAHD2a and FAHD2b proteins contain a mitochondrial pre-sequence (Table 1, marked by bold font), FAHD1 lacks such a pre-sequence (Table 1, marked in red), suggesting different mitochondrial import pathways for FAHD1 and FAHD2.

### 2.3 FAHD proteins are subject to differential mitochondrial import mechanisms

Proteins synthetized in the cytosol are imported into mitochondria *via* the general import pore (Lesnik et al., 2015; Walther and Rapaport, 2009), a multi-protein complex involving Tom5, Tom6, Tom7, Tom20, Tom22, Tom40, and Tom70. On the other hand, precursors of so-called signal-anchored proteins are imported to the mitochondria by a different mechanism (Ahting et al., 2005). Localization of FAHD1 in mitochondria despite the lack of a recognizable mitochondrial pre-sequence may suggest the presence of such a signal-anchor in FAHD1. The *UniProt* (Wasmuth and Lima, 2017) database lists curated (reviewed) entries of human proteins with signal-anchor motifs (keyword Signal-anchor KW-0735). *BLASTp* analysis of human FAHD1 and established signal anchor proteins displays significant sequence similarities with 8 entries, mapping to 4 proteins and their isoforms: Lactosylceramide *alpha-2,3-sialyltransferase* (Q9UNP4, Q9UNP4−2, Q9UNP4−3), *Beta-1,4-galactosyltransferase 7* (Q9UBV7), *Adipocyte plasma membrane-associated protein* (Q9HDC9, Q9HDC9−2), and *Membrane metallo-endopeptidase-like 1* (Q495T6, Q495T6−2). Alignment displays sequence identity in the amino acid ranges 1–24, 26–84, 27–131 and 185–207 of human FAHD1. For details on the dataset and computation see supplementary material.

This data may suggest a possible mechanism by which FAHD1 is synthetized in the cytosol and incorporated into mitochondria as a signal-anchored protein. The aforementioned predicted sites for cleavage of the FAHD1 sequence by MPP and ICP55 (see above) provide additional support for this theory. However, a possible cleavage by MPP at amino acids N26 and Y27 (see Table 1) would destroy the catalytic domain that is required for a functional protein (Weiss et al., 2018a) (see above), which appears unlikely. Hence, additional studies about processing of FAHD1 polypeptides during mitochondrial import seem warranted.

### 2.4 Potential interaction partners of FAHD proteins

Certain proteins have been listed in previous versions of the *BioPlex* (Huttlin et al., 2015) network, but have been removed in newer versions, probably reflecting a more stringent use of the COMPASS software (Huttlin et al., 2015) in more recent studies. Taking these changes into account, the probability of interaction partners may be ranked, preferring proteins that are listed in newer versions over proteins that were dropped in newer versions. Accordingly, the most probable binding partners of FAHD proteins are depicted as a bubble chart diagram in Fig. 2, each outer circle representing a lower ranking than the inner circles. The following proteins have been identified as potential FAHD1 interaction partners (see Fig. 2), some of which are also reported to interact with FAHD2:

*Carnitine palmitoyltransferase 2* (CPT2) is part of the carnitine shuttle system that is required for the import of palmitic acid into the mitochondrial matrix. CPT2 is localized at the matrix side of the inner mitochondrial membrane and required for the import of fatty acids into mitochondria (*UniProt* (Wasmuth and Lima, 2017)). *Clustered mitochondria homolog* (CLUH) is an mRNA-binding protein which is thought to ascertain proper cytoplasmic distribution of mitochondria. CLUH specifically binds mRNAs of nuclear-encoded mitochondrial proteins in the cytoplasm and regulates the transport and/or translation of these transcripts close to mitochondria, playing a role in mitochondrial biogenesis (*UniProt* (Wasmuth and Lima, 2017)). *NADH dependent ubiquinone oxidoreductase subunit S6* (NDUFS6) is an accessory subunit of the mitochondrial membrane respiratory chain *NADH dehydrogenase* (Complex I) (*UniProt* (Wasmuth and Lima, 2017)). *Polyribonucleotide nucleotidyltransferase 1* (PNPT1) as an RNA-binding protein is implicated in numerous RNA metabolic processes. It catalyzes the phosphorolysis of single-stranded polyribonucleotides processively in the 3′-5′ direction (*UniProt* (Wasmuth and Lima, 2017)). Putative *ubiquitin protein ligase E3 component n-recognin 3* (UBR3) is an E3 ubiquitin-protein ligase which is a component of the *N*-end rule pathway, leading to ubiquitination and subsequent degradation of its target proteins (*Uni-Prot* (Wasmuth and Lima, 2017)). *BolA family member 3* (BOLA3) acts as a mitochondrial iron-sulfur (Fe-S) cluster assembly factor that facilitates Fe-S cluster insertion into a subset of mitochondrial proteins (*UniProt* (Wasmuth and Lima, 2017)). *Heat shock protein family D (Hsp60) member 1* (HSPD1) is a chaperonin implicated in mitochondrial protein import and macromolecular assembly (*UniProt* (Wasmuth and Lima, 2017)).

Based on this dataset, we hypothesize a possible relation of FAHD proteins with fatty acid beta-oxidation and RNA metabolic processes. A possible association of FAHD1 with Complex I would support our model of FAHD1 acting as regulatory enzyme in the context of mitochondrial dysfunction associated senescence (MiDAS) described by us (Stöckl et al., 2006) and others (Wiley et al., 2016). However, more experimental data is required in order to probe for such connections.

## 3 FAHD proteins may play an unanticipated role in calcium homeostasis

### 3.1 Calcium in mitochondria

Calcium plays a key role in many vital processes, such as bone homeostasis, signal processing in neurons (inclusive serotonin effects), cell death and survival. Deterioration of calcium homeostasis is associated with aging (Herraiz-Martínez et al., 2015; Veldurthy et al., 2016), and both directly (Herraiz-Martínez et al., 2015) and indirectly linked to cholesterol homeostasis (van der Wulp et al., 2013; Wang et al., 2017). Serotonin levels and calcium homeostasis are linked to bone loss and type 2 diabetes (Erjavec et al., 2016). Vitamin D is associated to bone health and is an essential cofactor for calcium binding in the bone, which becomes even more important with aging (Veldurthy et al., 2016; Oudshoorn et al., 2009). The major calcium reservoir in cells is the endoplasmic reticulum. Mitochondrial calcium content is tightly regulated in most if not all eukaryotic cells.

Calcium uptake into and release from mitochondria is important in regulating a variety of cellular physiological functions (Takeuchi et al., 2015). Calcium handling by mitochondria is involved in energy production, in buffering and shaping cytosolic calcium, and in determining cell fate by triggering or preventing apoptosis (Contreras et al., 2010). Mitochondrial Ca^2+^ uptake is mainly mediated by a mitochondrial Ca^2+^ uniporter (MCU) driven by membrane potential (Perocchi et al., 2010), as well as by 2 H^+^ – Ca^2+^ exchange (Finkel et al., 2015). Mitochondrial Ca^2+^ is mainly released by a 3 Na^+^ – Ca^2+^ exchanger (NCLX) (Carafoli, 1974), but also by an active 2 H^+^ – Ca^2+^ exchange that has a dominant effect on release of Ca^2+^ from mitochondria in tissues in which mitochondrial NCLX activity is low (Takeuchi et al., 2015; Gunter and Pfeiffer, 1990). Calcium-binding mitochondrial carrier proteins (*e.g.* SLC25A12, SLC25A23, and SLC25A24) are reported to facilitate the calcium-dependent exchange of cytoplasmic metabolites across the mitochondrial inner membrane. However, there is scarce data on mitochondrial calcium binding proteins, except for *mitochondrial ATP synthase F1-beta-subunit* (Hubbard and McHugh, 1996), and for the predominantly mitochondrial protein *HAX1* (Balcerak et al., 2017).

Of note, uptake of Ca^2+^ requires co‐transport of an inner mitochondrial membrane permeable anion such as acetate or phosphate (Starkov, 2010), and the accumulated Ca^2+^ forms a detectable precipitate (Chinopoulos and Adam-Vizi, 2010) in the matrix of mitochondria in an apparently spontaneous process (Starkov, 2010). The granules contain significant amounts of carbon and nitrogen, indicating the presence of yet unidentified protein(s), that are suggested to serve as nucleation centers, facilitating formation of the Ca^2+^ precipitate (Starkov, 2010). This precipitate is suggested to be in pH equilibrium with the inner mitochondrial matrix, and eventually slowly released back into the cytosol (Starkov, 2010; Chinopoulos and Adam-Vizi, 2010).

During cellular activation Ca^2+^ levels in the mitochondrial matrix may reach up to μmol/L levels (Ivannikov and Macleod, 2013). High levels of intracellular Ca^2+^ activate mitochondrial *NADP dependent isocitrate dehydrogenase* (IDH2) and the *2-oxoglutarate dehydrogenase complex* (OGDC), as well as *pyruvate dehydrogenase phosphatase* (Pelley, 2007), which in turn activates the *pyruvate dehydrogenase complex* (PDC) (Pelley, 2007) to create acetyl-CoA to be used by *citrate synthase* (CS). These changes increase the reaction rate of many of the steps in the TCA cycle, and therefore increase flux throughout the pathway.

### 3.2 Endoplasmic reticulum and mitochondria direct the role of calcium in cellular senescence

Published links between calcium signaling and cellular senescence are summarized in a recent review by Martin and Bernard (Martin and Bernard, 2018), summarizing how calcium critically controls many molecular processes and cellular functions (Martin and Bernard, 2018; Humeau et al., 2018; Parys and Bultynck, 2018). In particular, knockdown of the mitochondrial calcium uniporter was reported to foster escape from senescence (Martin and Bernard, 2018). Elevation of intracellular calcium levels has been observed in response to different types of senescence-inducing stresses (telomere shortening, oncogene activation, rotenone or oxidative stress) in several cell types (Martin and Bernard, 2018). High concentrations of intracellular calcium are sustained during senescence (Martin and Bernard, 2018; Farfariello et al., 2015). This increase in calcium concentration has been attributed to calcium influx through plasma membrane calcium channels or to calcium release from the endoplasmic reticulum, depending on the context (Martin and Bernard, 2018; Giorgio et al., 2018). The endoplasmic reticulum was reported by many studies to play a key role in the regulation of calcium levels, cross-talking with mitochondria (Wiel et al., 2014; Gutiérrez and Simmen, 2018; Carreras-Sureda et al., 2018; Pitts and Hoffmann, 2018), *i.e.*, endoplasmic reticulum and mitochondria can be spatially and functionally coupled through mitochondria-associated endoplasmic reticulum membranes which favor the transfer of calcium from the endoplasmic reticulum to mitochondria (Patergnani et al., 2011). Endoplasmic reticulum chaperones tweak the mitochondrial calcium rheostat to control metabolism and cell death (Gutiérrez and Simmen, 2018). The main endoplasmic reticulum calcium release channels, *inositol 1,4,5-trisphosphate receptors* (ITPRs), were originally proposed as suppressors of autophagy (Bootman et al., 2018). In particular, calcium release through ITPR2 channels was reported to lead to mitochondrial calcium accumulation and senescence (Wiel et al., 2014). Calcium released from the endoplasmic reticulum in response to senescence-inducing stresses mainly exerts its effects through reactive oxygen species (Carreras-Sureda et al., 2018). In human mammary epithelial cells and primary human fibroblasts, oncogene activation and telomere shortening may also trigger calcium release from endoplasmic reticulum stores through the activation of the PLC/IP3/IP3R pathway (Martin and Bernard, 2018).

### 3.3 FAHD proteins are highly expressed in Ca^2+^ rich and Ca^2+^ regulating tissues

Calcium is the most abundant mineral in the human body, with Ca^2+^ concentration in plasma ranging between 2.1 and 2.6 mmol/L (Minisola et al., 2015), while higher calcium levels are defined as hypercalcemia (Minisola et al., 2015). While about 99 % of the body’s calcium is stored in the bone, about 1 % can be found in the blood serum, referred to as *free calcium*. The level of free calcium must remain within a very narrow concentration range to support vital physiological functions (Minisola et al., 2015). Cells absorb Ca^2+^ across the brush border of the enterocyte cell membrane by a mechanism that requires energy and *vitamin D* as an essential cofactor (Veldurthy et al., 2016), and *vitamin D* deficiency has been related to calcium homeostasis and aging (Oudshoorn et al., 2009; Kuro-o et al., 1997; Urakawa et al., 2006).

The absorption of calcium from food is performed by acid secretion from the stomach that converts calcium from various sources to Ca^2+^ salt which is then absorbed primarily in the duodenum. This mechanism is mainly influenced by conditions within the lumen of the small intestine. The thyroid gland releases calcitonin when levels of serum calcium are too high, which slows down the process of calcium release in the bone. The parathyroid gland produces parathyroid hormone when levels of serum calcium become too low, which in turn stimulates the release of calcium from the bones into the bloodstream. Hypocalcemia is mainly caused by malfunctions in the parathyroid gland. On the other hand, about 99 % of free calcium is reabsorbed by the kidney. Also, Ca^2+^ interferes with the absorption of iron (Fe^2+^) in the liver, so Ca^2+^ may accumulate in the liver (Kuchay, 2016). Of note, calcium homeostasis is highly important for the heart, and aging of the heart is associated with a decrease of calcium levels in the heart tissue (Herraiz-Martínez et al., 2015).

Table 4 summarizes the data on FAHD expression in human tissues, as listed in the *Human Protein Atlas* (Uhlen et al., 2005; Fagerberg et al., 2014; Uhlen et al., 2010). It is striking that FAHD1 is highly expressed in tissues that are associated with calcium metabolism and the regulation of calcium homeostasis. FAHD protein levels are generally high in the parathyroid gland, stomach, and kidney. FAHD1 levels are also high in the adrenal gland, small intestine and duodenum. Levels of FAHD2a and FAHD2b are high in the liver, thyroid gland and salivary gland, where levels of FAHD1 are high as well. There are several studies connecting these organs to calcium homeostasis and regulation (Brown and Vaidya, 2014; Ambudkar, 2016). The nasopharynx (displaying high levels of FAHD1) is usually not associated with calcium regulation, however, there is a recent documentation of a rare case of nasopharynx carcinoma because of hypercalcemia (Chaudhary and Sah, 2020). In contrast, detected FAHD protein levels are generally low in tissues that are not associated to calcium homeostasis Table 5.

### 3.4 Indirect evidence for calcium binding of FAHD proteins

*IonCom* (Zheng et al., 2019; Hu et al., 2016) analysis for human FAHD1 was performed to obtain information on predicted ion binding sites (see Table 4). This analysis was done by aligning deep neural-network based contact maps based on the 3D PDB structural data of human FAHD1 (6FOH). Potential binding sites have been predicted for Zn^2+^, Ca^2+^, Mg^2+^, Na^+^, K^+^, PO_4_^3^^−^. No binding sites have been predicted for Cu^2+^, Fe^2+/3+^, Mn^2+^, CO_3_^2-^, NO_2_^-^, SO_4_^2-^. The experimentally verified binding motif for Mg^2+^ in the catalytic domain (Weiss et al., 2018a) was successfully predicted by the algorithm. This is considered as a trustful quality control. Other binding sites are reported for Zn^2+^ and for Ca^2+^, as well as for PO_4_^3^^−^.

**Fig. 3:**
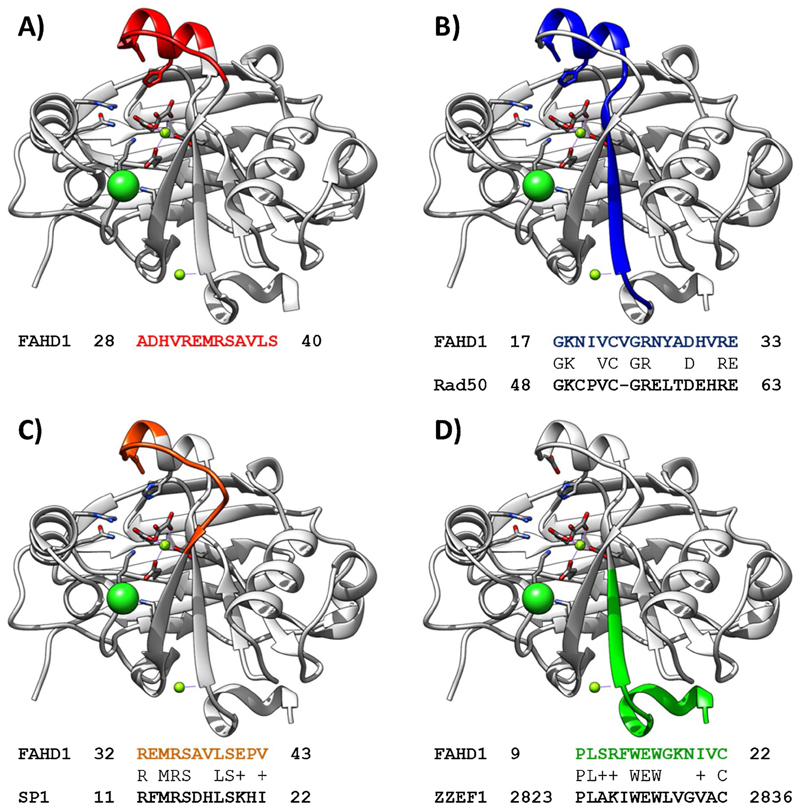
FAHD1 features sequence similarity with a human calcium-binding protein. *BLASTp* analysis of human FAHD1 was performed against reported Zn^**2+**^ and Ca^**2+**^ binding proteins. Individual structure motifs are displayed *via* coloring the tertiary structure of the PDB model 6FOG (Weiss et al., 2018a) of oxalate (OXL) complexed human FAHD1. Green spheres denote chloride ions in the dimerization site (Weiss et al., 2018a). Yellow spheres denote binding of bivalent metal ions, *i.e.*, Mg^**2+**^ in the PDB model 6FOG (Weiss et al., 2018a). **Panel A**: FAHD1 acquires catalytic competence through backbone-flip induced lid closure (Weiss et al., 2018a). This helical domain is displayed. **Panel B**: *BLASTp* analysis of human FAHD1 was performed against known Zinc binding proteins. Among the screened proteins, the *Rad50 coiled-coil Zn hook* (Hopfner et al., 2002) displays 53 % sequence identity (7 % sequence coverage) with human FAHD1 isoform 1 (*UniProt* (Wasmuth and Lima, 2017)-ID Q6P587). **Panel C**: *BLASTp* analysis of human FAHD1 was performed against known Zinc binding proteins. Among the screened proteins, the *Transcription Factor Sp1 DNA Binding Domain* (Oka et al., 2004) displays 50 % sequence identity (3 % sequence coverage) with human FAHD1 isoform 1 (*UniProt* (Wasmuth and Lima, 2017)-ID Q6P587). **Panel D**: *BLASTp* analysis of human FAHD1 was performed against known EF-hand domain-containing calcium-binding proteins (see text). Among the screened proteins, (only) human *Zinc* fi*nger ZZ-type and EF-hand domain-containing protein 1* (ZZEF1, *UniProt* (Wasmuth and Lima, 2017)-ID O43149) displays 43 % sequence identity (4 % sequence coverage) with human FAHD1 isoform 1 (*UniProt* (Wasmuth and Lima, 2017)-ID Q6P587). This reflects the finding of *IonCom* (Zheng et al., 2019; Hu et al., 2016) analysis for human FAHD1 (see Table 5).

Calcium-binding proteins participate in calcium cell signaling pathways by binding of calcium ions, thereby regulating the levels of free Ca^2+^ in the cytosol of the cell. Free calcium in the mitochondrial matrix can vary widely (100–800 nmol/L) (Finkel et al., 2015), depending on the extra-mitochondrial calcium level. Many different calcium-binding proteins exist, that are known to be heterogeneous, among them a group of proteins known as the EF-hand superfamily (Ishida and Vogel, 2013). The EF hand is a helix-loop-helix structural domain or motif found in a large family of calcium-binding proteins (Nakayama and Kretsinger, 1994). None of the reported EF-motifs (Ishida and Vogel, 2013) was fully identified in the sequence of FAHD1, but *BLASTp* analysis detected the amino acid sequence 142-DPHKLK-147 in FAHD1 that would partly match one of the reported EF-hand motifs (Ishida and Vogel, 2013) (SGREGDKHKLKKSE). *BLASTp* analysis of human FAHD1 was performed against known EF-hand domain-containing proteins (see Fig. 3D; see supplementary material for details on the dataset and computation). Among the screened proteins, human *Zinc* fi*nger ZZ-type and EF-hand domain-containing protein 1* (ZZEF1, *UniProt* (Wasmuth and Lima, 2017)-ID O43149) displays significant sequence identity with human FAHD1 isoform 1 (*UniProt* (Wasmuth and Lima, 2017)-ID Q6P587). The *N-* terminal motif is succeeded by a flexible loop region that is typical for FAH superfamily enzymes and participates in the catalytic mechanism (Weiss et al., 2018a) (see Fig. 3A). Allosteric regulation may be anticipated.

Similar data analysis has been performed for known zinc binding proteins, focusing on the *LIM domain* (PDB: 1X62), the *Zinc Finger 3* motif (PDB: 1VA3), the *coiled-coil Zn hook* (PDB: 1L8D) and *LCK fragments* (PDB: 1Q68). Among the four screened motifs, the *Zinc Finger 3* motif and the *coiled-coil Zn hook* showed significant sequence identity with FAHD1 in *BLASTp* analysis (see Fig. 3B and C). The two representative structures are *Zinc-hook domain-containing protein RAD50* (Hopfner et al., 2002) (see Fig. 3B) and *Transcription factor Sp1* (Oka et al., 2004) (see Fig. 3C). The Rad50 zinc-hook is a structure joining Mre11 complexes that are central to chromosomal maintenance, and functions in homologous recombination, telomere maintenance and sister chromatid association (Hopfner et al., 2002). SP1 is a transcription factor that can activate or repress transcription in response to physiological and pathological stimuli (Oka et al., 2004). It positively regulates the transcription of the core clock component ARNTL/BMAL1 (Oka et al., 2004) and plays an essential role in the regulation of FE65 gene expression (Oka et al., 2004). Albeit a local sequence similarity does not imply similar protein function in general, these data complement the data of possible FAHD1 interaction partners (see above) and contribute to the hypothesis of a potential relation of FAHD proteins with RNA metabolism.

The data of *IonCom* (Zheng et al., 2019; Hu et al., 2016) analysis suggesting Zn^2+^ and Ca^2+^ binding of FAHD1 seems to match with the *BLASTp* alignment of FAHD1 and zinc or calcium binding proteins, although no complete binding motif (ZZ-type, EF-hand, LIM domain, Zinc-hook, …) could be identified in the FAHD1 sequence.

**Fig. 4:**
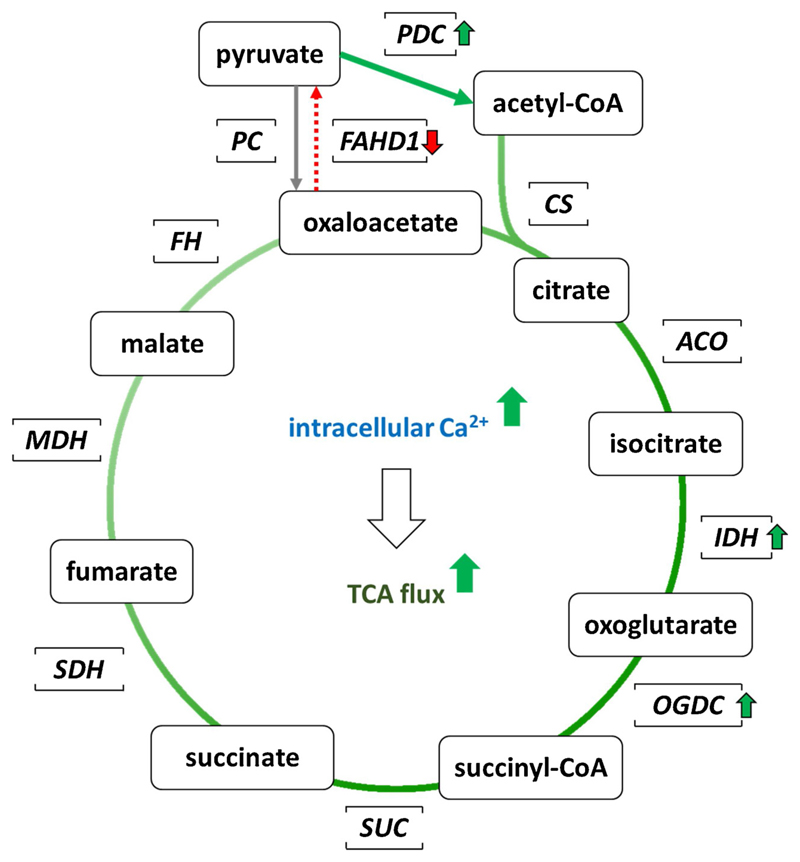
Increased intracellular Ca^**2+**^ levels generally increase the TCA flux and decrease FAHD1 activity in particular. During cellular activation Ca^**2+**^ levels in the mitochondrial matrix may reach up to μmol/L levels (Ivannikov and Macleod, 2013). This is associated to a general increase of the TCA flux, in particular to an activation of *NADP dependent isocitrate dehydrogenase* (IDH2) and *2-oxoglutarate dehydrogenase* (OGDH, as part of the OGDC complex) (Denton et al., 1975). Of note, increased Ca^**2+**^ levels also activate *pyruvate dehydrogenase phosphatase* (Pelley, 2007), which in turn activates the *pyruvate dehydrogenase complex* (PDC) (Pelley, 2007) to create acetyl-CoA to be used by *citrate synthase* (CS). We propose a model where FAHD1 is regulated by a contest of cofactor Mg^**2+**^ and competing Ca^**2+**^ ions, and its catalytic ODx activity is decreased by increased Ca^**2+**^ levels (see text and Fig. 4). On the other hand, decreased Ca^**2+**^ levels would decrease oxaloacetate levels by activation of FAHD1.

FAHD1 shows highest ApH-activity with Mg^2+^ and Mn^2+^ as cofactors, whereas Ca^2+^- and Zn^2+^-bound enzyme displays strongly reduced catalytic activity (Pircher et al., 2011). ODx activity of FAHD1 prefers the same metals as ApH. Such findings implicate that distinct divalent metal ions, such as Ca^2+^ and Zn^2+^, may be prone to inhibit the catalytic activity of FAH superfamily proteins. High levels of calcium would reduce FAHD1’s enzymatic activity by contest of cofactor Mg^2+^ and competing Ca^2+^ ions. We further tested if there is a potential contest of the cofactors that may be associated to Ca^2+^ regulation. When catalytic activity of recombinant human FAHD1 (Weiss et al., 2019) was tested in *in vitro* assays against cofactor concentrations, we observed a significant decrease of ODx activity with increasing Ca^2+^ concentrations (A. Weiss et al., unpublished). We propose a model where FAHD1 is regulated by a contest of cofactor Mg^2+^ and competing Ca^2+^ ions, and its catalytic ODx activity is decreased by increased Ca^2+^ levels (see Fig. 4). In consequence, decreased Ca^2+^ levels would decrease oxaloacetate levels by activation of FAHD1 (in the presence of Mg^2+^).

### 3.5 FAHD1 effects on serotonin signaling – a link to Ca^2+^ signaling?

We could show that egg laying behavior is altered in *fahd-1* depleted *Caenorhabditis elegans* (Taferner et al., 2015; Baraldo et al., 2019). Whereas wild-type animals do not lay eggs when put in a hypertonic salt solution and commence egg-laying only after serotonin-treatment, *fahd-1* (-/-) worms did not cease egg-laying under these unfavorable conditions (Taferner et al., 2015; Baraldo et al., 2019) nor did they increase their egg-laying rate upon contact with exogenously applied serotonin (up to 10 mM) (Baraldo et al., 2019). It is known that egg-laying is an active process which is regulated by neuronal signals mediated by serotonin (and several other neurotransmitters) (Horvitz et al., 1982; Trent et al., 1983) and requires intact vulval musculature (Desai et al., 1988; Schinkmann and Li, 1992; Weinshenker et al., 1995). Altered egg-laying behavior in *fahd-1* depleted worms was associated with a significant upregulation of the gene *basl-1*, that is predicted to have carboxylyase activity and *pyridoxal phosphate* binding activity (WormBase, WBGene00015467#0−9f-10). *BLASTp* analysis of *UniProt* (Wasmuth and Lima, 2017) entry O45138 *BAS-Like OS=Caenorhabditis elegans* provided about 35 % sequence identity with *UniProt* (Wasmuth and Lima, 2017) entry P20711, the human protein *aromatic-* L-*amino-acid decarboxylase* (DDC, also PXLP-DDC or AADC). This protein catalyzes the decarboxylation of *L-dopa* to dopamine, and of *5-hydroxy*-L-*tryptophan* to serotonin (EC:4.1.1.28). The catalytic activity of the human protein matches the reported activity of the nematode protein. Upregulation of *basl-1* as a reaction to *fahd-1* knockout would, therefore, indicate the increased production of serotonin from precursor metabolite *5-hydroxy*-L-*tryptophan*. From these data we concluded that FAHD-1 in *Caenorhabditis elegans* modulates serotonin signaling (Baraldo et al., 2019).

Calcium homeostasis in nematodes is involved in movement, fertility, egg-laying and growth of *Caenorhabditis elegans* (Bandyopadhyay et al., 2002), and it may in fact be a deteriorated calcium homeostasis that impacts the nematode’s egg-laying behavior, as was implied by others (Bandyopadhyay et al., 2002). Recent work on serotonin signaling and calcium homeostasis in different species showed diverse outcomes. Effects have been reported in studies of milk production and milk quality in dairy cows (Hernández-Castellano et al., 2017; Weaver et al., 2016), where a certain ambiguity between cause and relation of serotonin and calcium homeostasis is described. Serotonin is mainly responsible for increasing calcium pumps in the mammary gland (Hernandez et al., 2012) and secretion into milk (Laporta et al., 2013). Infusion of serotonin acutely decreased free calcium concentrations Weaver et al., 2016) in dairy cows, while also decreasing calcium excretion in urine and increasing calcium levels in milk (Laporta et al., 2013). This is in contrast to other work with rats, where elevated blood serotonin is associated with increased levels of free calcium concentrations (Erjavec et al., 2016) because of bone loss and the development of type 2 diabetes (Erjavec et al., 2016). It is discussed that a possible answer to this problem might be the explanation of a time-dependent change in metabolism, where an acute change in serotonin (such as feeding serotonin to cows for days) differs from a long-term change in metabolism (such as rats with long term inhibitory treatment). In *Caenorhabditis elegans*, calcium imaging studies could show that serotonin acts directly on the vulval muscles to increase the frequency of spontaneous calcium transients, thus increasing egg-laying (Shyn et al., 2003).

Current data reveals a link of FAHD-1 depletion in *Caenorhabditis elegans* to a significant change in the nematode’s serotonin signaling pathway. However, more elaborate experiments on serotonin signaling and calcium homeostasis in *Caenorhabditis elegans* are warranted to reveal a possible link to FAHD-1 depletion.

## 4 Discussion and outlook

### 4.1 Multiple physiological functions of FAHD proteins in eukaryotes

Predicted protein interaction partners of FAHD1 reflect its reported localization (Pircher et al., 2011; Wasmuth and Lima, 2017; Uhlen et al., 2010), and suggest a putative role of FAHD proteins in the pathways of fatty acid oxidation, oxidative phosphorylation, mitochondrial RNA metabolism and the ubiquitin/proteasome system. As available data from high-throughput proteomics analysis (Huttlin et al., 2015) suggest, the most probable interaction partners of FAHD1 are *carnitine palmitoyltransferase 2* (CPT2), *clustered mitochondria homolog* (CLUH), *NADH dependent ubiquinone oxidoreductase subunit S6* (NDUFS6), *polyribonucleotide nucleotidyltransferase 1* (PNPT1), and *putative ubiquitin protein ligase E3 component n-recognin 3* (UBR3). NDUFS6 is an accessory subunit of the mitochondrial membrane respiratory chain complex I. A putative interaction with FAHD1 may complement our recently hypothesized model of senescence (Etemad et al., 2019) due to the inactivation of genes required for mitochondrial function (such as SIRT3 (Hallows et al., 2011) and FAHD1 (Etemad et al., 2019)), thus explaining how in some cellular models the inactivation of either ETC complex I (by metformin) or ETC complex II (by FAHD1 knockdown) has the potential to increase *p21* gene expression in the absence of AMPK (Etemad et al., 2019). In agreement with results obtained from a high-throughput proteomics study (Dittenhafer-Reed et al., 2015), we recently provided circumstantial evidence for a SIRT3 deacetylation site (Dittenhafer-Reed et al., 2015) in mouse FAHD1 (Weiss et al., 2020), which further supports this model.

### 4.2 A new role for FAHD1 in calcium homeostasis?

FAHD proteins are members of the FAH superfamily of metabolic enzymes, the physiological role of which is only partially explored. In the case of FAHD1, existing evidence suggests that it is a mitochondrial protein which can catalyze hydrolysis of acylpyruvates and the decarboxylation of oxaloacetate. However, several features of FAHD1 activity remain largely unexplored, in particular due to the fact that experiments with FAHD1/2 depleted cells and animals still lack considerable mechanistic detail. The main purpose of this review is to stimulate discussions in this understudied field of research, and to critically review the research agenda how to unmask molecular mechanisms of action for these proteins.

We have proposed a model of how FAHD1 catalytic activity as oxaloacetate decarboxylase in mitochondria may describe FAHD1 as a regulator of TCA cycle flux and as a possible regulator of mitochondrial function and senescence (Etemad et al., 2019). We now propose a complementary model of how the actual presence of FAHD1 protein (or lack thereof), independent of its catalytic function, may influence intracellular calcium levels. It is well reported that FAHD1 expression in human organs correlates with the regulation of calcium metabolism in the human body, and experimental results described in this work are in line with the hypothesis that FAHD1 may be a calcium binding protein. Calcium binding proteins are present in various cellular compartments and serve to mediate effects of increased calcium concentration on biological responses. On the other hand, it is conceivable that calcium binding proteins serve as buffering systems to fine-tune the concentration of intracellular calcium. Our unpublished observation that increasing levels of calcium inactivate FAHD1 catalytic activity *in vitro* is in line with the model of how calcium levels modulate the TCA cycle flux (Etemad et al., 2019) (Fig. 4). The model predicts coordinated but inverse regulation of FAHD1 and the canonical TCA cycle enzymes IDH and OGDC, respectively, suggesting a regulatory mechanism by which increasing calcium levels in the mitochondrial matrix booster flux through the TCA cycle.

## Supplementary Material

Supplementary material related to this article can be found, in the online version, at doi:https://dx.doi.org/10.1016/j.mad.2020.111284.

suppl. files SF1-SF3, suppl. figures S1-S3

## Figures and Tables

**Table 1:** A survey of predicted mitochondrial targeting sequences and their cleavage sites using the MitoFates (Fukasawa et al., 2015) server. FAHD1 is not predicted to have a mitochondrial pre-sequence (marked in red), but the FAHD2a and FAHD2b sequences are. All listed enzymes display a site for proteolytic cleavage of the targeting signal, performed by the mitochondrial processing peptidase (MPP). All FAHD proteins display a site for cleavage of destabilizing N-terminal amino acid residues by intermediate cleaving peptidase 55 (ICP55), which is critical for stabilization of the mitochondrial proteome (Wasmuth and Lima, 2017) (see also Figure S2).

Enzyme	*Uni* Prot-Spec	Probability of pre-sequence	Mitochondrial pre-sequence	Cleavage site	Positions for TOM20 recognition motifs

CS	CISY_HUMAN	0.996	yes	25(MPP)	7-11

ACO	ACONJHUMAN	0.995	yes	19(MPP)	11-15

IDH2	IDHPJHUMAN	0.993	yes	38(MPP), 39(lcp55)	4-8,58-62

	IDH3A_HUMAN	0.961	yes	26(MPP), 27(lcp55)	10-14,50-54
IDH3	DH3BJHUMAN	0.997	yes	25(MPP), 33(Octl)	10-14,31-35,63-67,70-74
	IDH3G_HUMAN	0.801	yes	38(MPP)	2-6,12-16,77-81

	ODOIJHUMAN	0.996	yes	39(MPP), 40(lcp55)	
OGDC	OD02HUMAN	0.999	yes	59(MPP), 67(Octl)	8-12,89-93
	DLDHJHUMAN	0.996	yes	34(MPP), 35(lcp55)	4-8,57-61

	SUCAJHUMAN	0.421	yes	40(MPP)	23-27
SUC (A/G)	SUCB2_HUMAN	0.964	yes	22(MPP), 23(lcp55)	9-13,12-16,56-60
	SUCB1_HUMAN	0.826	yes	52(MPP), 53(lcp55)	7-11,24-28

	SDHAJHUMAN	0.995	yes	32(MPP), 40(Octl)	7-11,13-17,18-22,90-94
	SDHB HUMAN	0.963	yes	28(MPP)	39-43
SDH	C560JHUMAN	0.992	yes	51(MPP), 52(lcp55)	38-42
	DHSD_HUMAN	0.996	yes	28(MPP)	

FH	FUMHJHUMAN	1.000	yes	44(MPP)	1-5,4-8,41-45,92-96

MDH2	MDHMJHUMAN	0.999	yes	16(MPP), 24(Octl)	

FAHD1	FAHD1JHUMAN	0.123	no	26(MPP), 27(lcp55)	10-14

FAHD2a	FAH2AJHUMAN	0.790	yes	83(MPP), 84(lcp55)	34-38,80-84

FAHD2b	FAH2BJHUMAN	0.884	yes	83(MPP), 84(lcp55)	34-38,80-84

**Table 2:** A survey of mitochondrial TCA cycle enzymes, comparing the reported structure and predicted stability in solution at physiological conditions. Structure and general protein information has been obtained from the UniProt (Wasmuth and Lima, 2017) database. Theoretical pi and stability predictions have been computed using the ProtParam server (Gasteiger et al., 2005). FAHD proteins are predicted to be unstable (marked in red), however, FAHD1 is understood to form a soluble and catalytic active homodimer (Pircher et al., 2011, 2015; Weiss et al., 2018a; Manjasetty et al., 2004), whereas all other unstable proteins are part of greater protein complexes (Wasmuth and Lima, 2017) (marked in green). Protein interaction of FAHD1 is likely (Huttlin et al., 2015) (see Table 3). The protein structure of FAHD2a and FAHD2b is yet unreported.

Enzyme	Name	UniProt-lD	UniProt- Spec	Theoretical pi	Instability index	Stability	Part of a complex	Structure

CS	Citrate synthase	075390	CISYJHUMAN	8.45	22.40	stable	no	homodimer

ACO	Aconitate hydratase	Q99798	ACON_HUMAN	7.36	34.70	stable	no	monomer

IDH2	Isocitrate dehydrogenase [NADP]	P48735	IDHPJHUMAN	8.88	29.77	stable	no	homodimer

	Isocitrate dehydrogenase [NAD] subunit alpha	P50213	IDH3AJHUMAN	6.46	41.24	unstable	yes (IDH3)	complex
IDH3	Isocitrate dehydrogenase [NAD] subunit beta	043837	DH3BJHUMAN	8.64	36.88	stable	yes (IDH3)	complex
	Isocitrate dehydrogenase [NAD] subunit gamma	P51553	IDH3GJHUMAN	8.75	45.59	unstable	yes (IDH3)	complex

	2-oxoglutarate dehydrogenase	Q02218	ODOIJHUMAN	6.39	45.17	unstable	yes (OGDC)	complex
OGDC	Dihydrolipoyllysine-residue succinyltransferase	P36957	0D02JHUMAN	9.10	50.53	unstable	yes (OGDC)	complex
	Dihydrolipoyl dehydrogenase	P09622	DLDH_HUMAN	7.95	28.07	stable	yes (OGDC)	complex

	Succinate--CoA ligase [ADP/GDP-forming] subunit alpha	P53597	SUCAJHUMAN	9.01	41.30	unstable	yes (SUCA/SUCG)	complex
SUC(A/G)	Succinate--CoA ligase [GDP-forming] subunit beta	Q96I99	SUCB2HUMAN	6.15	32.54	stable	yes (SUCG)	complex
	Succinate--CoA ligase [ADP-forming] subunit beta	Q9P2R7	SUCB1JHUMAN	7.05	41.13	unstable	yes (SUCA)	complex

	Succinate dehydrogenase [ubiquinone] flavoprotein subunit	P31040	SDHAJHUMAN	7.06	37.04	stable	yes (SDH)	complex
SDH	Succinate dehydrogenase [ubiquinone] iron-sulfur subunit	P21912	SDHBJHUMAN	9.03	60.13	unstable	yes (SDH)	complex
	Succinate dehydrogenase cytochrome b560 subunit	Q99643	C560JHUMAN	9.74	47.79	unstable	yes (SDH)	complex
	Succinate dehydrogenase [ubiquinone] cytochrome b small subunit	014521	DHSD_HUMAN	8.92	33.20	stable	yes (SDH)	complex

FH	Fumarate hydratase	P07954	FUMH_HUMAN	8.85	28.59	stable	no	homotetramer

MDH2	Malate dehydrogenase	P40926	MDHM_HUMAN	8.92	31.92	stable	no	homodimer

FAHD1	Fumarylacetoacetate hydrolase domain containing protein 1	Q6P587	FAHD1_HUMAN	6.96	42.36	unstable	likely	homodimer

FAHD2a	Fumarylacetoacetate hydrolase domain containing protein 2a	Q96GK7	FAH2A_HUMAN	8.48	41.26	unstable	unknown	unknown

FAHD2b	Fumarylacetoacetate hydrolase domain containing protein 2b	Q6P2I3	FAH2B_HUMAN	7.64	40.43	unstable	unknown	unknown

**Table 3:** Potential interaction partners of FAHD proteins, as listed in the BioPlex (Huttlin et al., 2015) network of different versions. Highlighted in gray are proteins that are listed in the newest versions 2 and 3 of the network. Other proteins were listed in early versions of the network but removed in the latest stable version 3. Localization and description of the proteins was gathered from the Human Protein Atlas (Uhlen et al., 2005; Fagerberg et al., 2014; Uhlen et al., 2010) and the UniProt (Wasmuth and Lima, 2017) database.

Enzyme	Interaction	BioPlex	Localization (Human Protein Atlas, UniProt)	Description

	ARL2	3.0	Nucleoplasm, Nucleoli, Golgi apparatus, Focal adhesion sites, Cytosol	ADP ribosylation factor like GTPase 2
	PTRHD1	3.0	Nucleoplasm	Peptldyl-tRNA hydrolase domain containing 1
	CPT2	3.0, 2.0, 1.0	Nucleoplasm, Nucleoli, Mitochondria	Carnitine palmitoyltransferase 2
	DHRS4	3.0	Vesicles, Nuclear membrane	Dehydrogenase/reductase 4
	DHTKD1	2.0, 1.0	Mitochondria	Dehydrogenase El and transketolase domain containing 1
	FSD1	2.0, 1.0	Nucleus	Flbronectin type III and SPRY domain containing 1
FAHD1	INHA	3.0	Vesicles	Inhibin alpha subunit
	CLUH	3.0, 2.0, 1.0	Vesicles, Nuclear bodies	Clustered mitochondria homolog
	MTERFD1	3.0	Nucleoplasm, Mitochondria	Mitochondrial transcription termination factor 3
	NDUFS6	3.0, 2.0	Mitochondria	NADH:ublquinone oxldoreductase subunit S6
	OR10H3	2.0, 1.0	Cell membrane	Olfactory receptor family 10 subfamily H member 3
	PNPT1	3.0, 2.0, 1.0	Mitochondria	Polyribonucleotide nucleotidyltransferase 1
	UBR3	3.0, 2.0, 1.0	Nucleoplasm, Nucleoli	Ubiquitln protein llgase E3 component n-recognin 3 (putative)

	BOLA3	3.0, 2.0, 1.0	Nuclear bodies, Mitochondria, Cytosol	BolA family member 3
	DBT	3.0, 2.0, 1.0	Mitochondria	Dlhydrolipoamide branched chain transacylase E2
	FAHD2B	3.0, 2.0	unreported	Fumarylacetoacetate hydrolase domain containing 2B
	FCER1A	3.0	Cell membrane	Fc fragment of IgE receptor la
	FCGR2A	2.0	Plasma membrane, Golgi apparatus	Fc fragment of IgG receptor lia
FAHD2A	LCN8	3.0	Secreted	Lipocalin 8
	OMP	3.0	Cytoplasm	Olfactory marker protein
	SETX	3.0	Nucleoplasm, Cytokinetic bridge	Senataxin
	SOX2	3.0, 2.0, 1.0	Nucleoplasm	SRY-box2
	TAS2R41	2.0	Cell membrane	Taste 2 receptor member 41
	ZNF287	3.0	Golgi apparatus	Zinc finger protein 287

	BOLA3	3.0	Nuclear bodies, Mitochondria, Cytosol	BolA family member 3
	DBT	3.0	Mitochondria	Dlhydrolipoamide branched chain transacylase E2
FAHD2B	FAHD2A	3.0	unreported	Fumarylacetoacetate hydrolase domain containing 2A
	FCGR2A	1.0	Plasma membrane, Golgi apparatus	Fc fragment of IgG receptor lia
	HSPD1	3.0, 2.0	Mitochondria	Heat shock protein family D (Hsp60) member 1

**Table 4:** Expression levels (high, medium, low) of FAHD protein (not mRNA levels) in human organs, according to the data listed in the Human Protein Atlas (Uhlen et al., 2005; Fagerberg et al., 2014; Uhlen et al., 2010). In particular, FAHD1 is highly expressed in organs that are associated to the regulation of calcium metabolism and of calcium homeostasis.

	Protein expression (Human Protein Atlas)			

Regulatory role in human Ca^2+^ metabolism	human organ	FAHDl	FAHD2a	FAHD2b

major control unit of the body’s calcium levels	Parathyroid gland	high	high	high
Ca^2+^ uptake from food	Stomach	high	high	high
major Ca^2t^ resorption from blood	Kidney	high	high	high
regulation of Ca^2+^ homeostasis	Adrenal gland	high	high	medium
Hypercalcemia reported for rare nasopharynx carcinoma	Nasopharynx	high	high	medium
major modulation unit of Ca^2+^ absorption	Small intestine	high	high	medium
primary Ca^2+^ absorption	Duodenum	high	medium	medium
secondary Ca^2+^ absorption	Colon	high	medium	medium
	Rectum	high	medium	medium
	Gallbladder	high	medium	medium
	Seminal vesicle	high	medium	medium
	Endometrium	high	medium	medium
	Appendix	high	medium	low
	Urinary bladder	high	low	low

serum Ca^2+^ sensitive stimulation the parathyroid gland	Thyroid gland	medium	high	high
Ca^2+^ is a critical factor in control of salivary gland function	Sailvary gland	medium	high	high
Ca^2+^ levels modulate the Iron homeostasis In the liver	Liver	medium	high	high
	Testis	medium	high	high
	Bronchus	medium	high	medium
	Cerebral Cortex	medium	medium	medium
	Pancreas	medium	medium	medium
	Epididymis	medium	medium	medium
	Fallopian tube	medium	medium	medium
	Breast	medium	medium	medium
	Heart muscle	medium	medium	medium
	Cervix, uterine	medium	medium	low
	Cerebellum	medium	low	medium
	Lung	medium	low	medium
	Esophagus	medium	low	low
	Prostate	medium	low	low
	Placenta	medium	low	low
	Skin	medium	low	low
	Torsil	medium	low	…
	Vagina	medium	…	…

	Hippocampus	low	medium	medium
	Caudate	low	medium	medium
	Soft tissue	low	low	medium
	Bone marrow	low	low	low
	Oral mucosa	low	…	…
	Spleen	low	…	…

…	Skeletal muscle		medium	medium
…	Smooth muscle		low	medium
	Ovary	…	…	…
	Adipose tissue	…	…	…
	Lymph node	…	…	…

**Table 5:** Ion ligand binding prediction using the *IonCom* (Zheng et al., 2019; Hu et al., 2016) analysis, by aligning deep neural-network based contact maps based on the PDB data of human FAHD1 (6FOH). Potential binding sites have been predicted for Zn^2^+, Ca^2^+, Mg^2^+, Na+, K+, PO4^3-^, No binding sites have been predicted for Cu^2+^, Fe^2+/3+^, Mn^2+^, CO_3_^2-^, NO_2_’, SO_4_^2-^.

	Zn^2+^	Ca^2+^	Mg^2+^	Na^+^	K^+^	PO_4_^3-^
G17						
K18							
C22						
V23						
G24						
R25						
S36						
F45						
S49						
E55						
H69						
E71						
E73						
C82						
V85						
Y97						
L1Ol						
D102						
M103						
R106						
D107						
Q109						
C112						
W119						
K123						
F125						
T126						
C129						
S132						
L150						
N153						
E155						
E159						
D186						
G191						
T192						
D203						
E204						
1205						
A207						
S214						
E223						

## References

[R1] Ahting U, Waizenegger T, Neupert W, Rapaport D (2005). Signal-anchored proteins follow a unique insertion pathway into
the outer membrane of mitochondria. J Biol Chem.

[R2] Almagro Armenteros JJ, Salvatore M, Emanuelsson O, Winther O, von Heijne G, Elofsson A (2019). Detecting sequence signals in targeting peptides using deep
learning. Life Sci Alliance.

[R3] Ambudkar IS (2016). Calcium signalling in salivary gland physiology and
dysfunction. J Physiol.

[R4] Anna B, Wojtczak EW (1974). Mitochondrial oxaloacetate decarboxylase from rat
liver. Biochim Biophys Acta.

[R5] Balcerak A, Rowinski S, Szafron LM, Grzybowska EA (2017). The calcium binding properties and structure prediction of the
Hax-1 protein. Acta Biochim Pol.

[R6] Bandyopadhyay J, Lee J, Lee J, Lee JIl, Yu J-R, Jee C (2002). Calcineurin, a calcium/calmodulin-dependent protein phosphatase,
is involved in movement, fertility, egg laying, and growth in Caenorhabditis
elegans. Mol Biol Cell.

[R7] Baraldo G, Etemad S, Weiss AKH, Jansen-Dürr P, Mack HID (2019). Modulation of serotonin signaling by the putative oxaloacetate
decarboxylase FAHD-1 in Caenorhabditis elegans. PLoS One.

[R8] Bateman RL, Bhanumoorthy P, Witte JF, McClard RW, Grompe M, Timm DE (2001). Mechanistic inferences from the crystal structure of
fumarylacetoacetate hydrolase with a bound phosphorus-based
inhibitor. J Biol Chem.

[R9] Bootman MD, Chehab T, Bultynck G, Parys JB, Rietdorf K (2018). The regulation of autophagy by calcium signals: do we have a
consensus?. Cell Calcium.

[R10] Brown JM, Vaidya A (2014). Interactions between adrenal-regulatory and calcium-regulatory
hormones in human health. Curr Opin Endocrinol Diabetes Obes.

[R11] Carafoli E (1974). The release of calcium from heart mitochondria by
sodium. J Mol Cell Cardiol.

[R12] Carreras-Sureda A, Pihán P, Hetz C (2018). Calcium signaling at the endoplasmic reticulum: fine-tuning
stress responses. Cell Calcium.

[R13] Chaudhary S, Sah JP (2020). Hypercalcemia due to Nasopharyngeal Carcinoma. JNMA J Nepal Med Assoc.

[R14] Chinopoulos C, Adam-Vizi V (2010). Mitochondrial Ca2+ sequestration and precipitation
revisited. FEBS J.

[R15] Contreras L, Drago I, Zampese E, Pozzan T (2010). Mitochondria: The calcium connection. Biochim Biophys Acta (BBA) - Bioenergetics.

[R16] Corwin LM (1959). Oxaloacetic decarboxylase from rat liver
mitochondria. J Biol Chem.

[R17] Denton RM, Randle PJ, Bridges BJ, Cooper RH, Kerbey AL, Pask HT (1975). Regulation of mammalian pyruvate dehydrogenase. Mol Cell Biochem.

[R18] Desai C, Garriga G, McIntire SL, Horvitz HR (1988). A genetic pathway for the development of the Caenorhabditis
elegans HSN motor neurons. Nature.

[R19] Dittenhafer-Reed KE, Richards AL, Fan J, Smallegan MJ, Fotuhi Siahpirani A, Kemmerer ZA (2015). SIRT3 mediates multi-tissue coupling for metabolic fuel
switching. Cell Metab.

[R20] Dorigatti I, Weiss AKH, Jansen-Dürr P (2018). Purification of N-terminal Truncated Variants of Human
Fumarylacetoacetate Hydrolase Domain Containing Protein 2a (hFAHD2a);
Bachelor Thesis in Biology. Leopold-Franzens-University Innsbruck.

[R21] Erjavec I, Bordukalo-Niksic T, Brkljacic J, Grcevic D, Mokrovic G, Kesic M (2016). Constitutively elevated blood serotonin is associated with bone
loss and type 2 diabetes in rats. PLoS One.

[R22] Etemad S, Petit M, Weiss AKH, Schrattenholz A, Baraldo G, Jansen-Dürr P (2019). Oxaloacetate decarboxylase FAHD1 – a new regulator of
mitochondrial function and senescence. Mech Ageing Dev.

[R23] Fagerberg L, Hallström BM, Oksvold P, Kampf C, Djureinovic D, Odeberg J (2014). Analysis of the human tissue-specific expression by genome-wide
integration of transcriptomics and antibody-based proteomics. Mol Cell Proteom.

[R24] Farfariello V, Iamshanova O, Germain E, Fliniaux I, Prevarskaya N (2015). Calcium homeostasis in cancer: a focus on
senescence. Biochim Biophys Acta (BBA) – Mol Cell Res.

[R25] Finkel T, Menazza S, Holmström KM, Parks RJ, Liu JJ, Sun J (2015). The ins and outs of mitochondrial calcium. Circ Res.

[R26] Fukasawa Y, Tsuji J, Fu S-C, Tomii K, Horton P, Imai K (2015). MitoFates: improved prediction of mitochondrial targeting
sequences and their cleavage sites. Mol Cell Proteom.

[R27] Gasteiger E, Hoogland C, Gattiker A, Duvaud S, Wilkins MR, Appel RD, B A (2005). Protein identification and analysis tools on the ExPASy
server. In: Walker, John M. (Ed.), The Proteomics Protocols Handbook. Humana
Press.

[R28] Giorgio V, Guo L, Bassot C, Petronilli V, Bernardi P (2018). Calcium and regulation of the mitochondrial permeability
transition. Cell Calcium.

[R29] Groebe K, Krause F, Kunstmann B, Unterluggauer H, Reifschneider NH, Scheckhuber CQ (2007). Differential proteomic profiling of mitochondria from Podospora
anserina, rat and human reveals distinct patterns of age-related oxidative
changes. Exp Gerontol.

[R30] Gunter TE, Pfeiffer DR (1990). Mechanisms by which mitochondria transport
calcium. Am J Physiol-Cell Physiol.

[R31] Gutiérrez T, Simmen T (2018). Endoplasmic reticulum chaperones tweak the mitochondrial calcium
rheostat to control metabolism and cell death. Cell Calcium.

[R32] Hallows WC, Yu W, Smith BC, Devires MK, Ellinger JJ, Someya S (2011). Sirt3 promotes the urea cycle and fatty acid oxidation during
dietary restriction. Mol Cell.

[R33] Hernandez LL, Gregerson KA, Horseman ND (2012). Mammary gland serotonin regulates parathyroid hormone-related
protein and other bone-related signals. Am J Physiol-Endocrinol Metab.

[R34] Hernández-Castellano LE, Hernandez LL, Weaver S, Bruckmaier RM (2017). Increased serum serotonin improves parturient calcium homeostasis
in dairy cows. J Dairy Sci.

[R35] Herraiz-Martínez A, Álvarez-García J, Llach A, Molina CE, Fernandes J, Ferrero-Gregori A (2015). Ageing is associated with deterioration of calcium homeostasis in
isolated human right atrial myocytes. Cardiovasc Res.

[R36] Holzknecht M, Weiss AKH, Jansen-Dürr P (2018). A Conjunct Study of FAH-domain Containing Proteins: Expression,
Purification and Characterization of hFAHD2a; Master Thesis in
Biology. Leopold-Franzens University of Innsbruck.

[R37] Hong H, Seo H, Park W, Kim K (2020). Sequence, structure and function‐based classification of
the broadly conserved FAH superfamily reveals two distinct fumarylpyruvate
hydrolase subfamilies. Environ Microbiol.

[R38] Hopfner K-P, Craig L, Moncalian G, Zinkel RA, Usui T, Owen BAL (2002). The Rad50 zinc-hook is a structure joining Mre11 complexes in DNA
recombination and repair. Nature.

[R39] Horvitz HR, Chalfie M, Trent C, Sulston JE, Evans PD (1982). Serotonin and octopamine in the nematode Caenorhabditis
elegans. Science (New York, N.Y.).

[R40] Hu X, Dong Q, Yang J, Zhang Y (2016). Recognizing metal and acid radical ion-binding sites by
integrating ab initio modeling with template-based
transferals. Bioinformatics (Oxford, England).

[R41] Hubbard MJ, McHugh NJ (1996). Mitochondrial ATP synthase F 1 -β-subunit is a
calcium-binding protein. FEBS Lett.

[R42] Humeau J, Bravo-San Pedro JM, Vitale I, Nuñez L, Villalobos C, Kroemer G (2018). Calcium signaling and cell cycle: progression or
death. Cell Calcium.

[R43] Huttlin EL, Ting L, Bruckner RJ, Gebreab F, Gygi MP, Szpyt J (2015). The BioPlex network: a systematic exploration of the human
interactome. Cell.

[R44] Ishida H, Vogel HJ (2013). EF-hand proteins. Encyclopedia of Metalloproteins.

[R45] Ivannikov MV, Macleod GT (2013). Mitochondrial free Ca^2+^ levels and their effects on
energy metabolism in Drosophila motor nerve terminals. Biophys J.

[R46] Jansen-Duerr P, Pircher H, Weiss AKH (2016). The FAH fold meets the krebs cycle. Mol Enzymol Drug Targets.

[R47] Kang T-W, Yevsa T, Woller N, Hoenicke L, Wuestefeld T, Dauch D (2011). Senescence surveillance of pre-malignant hepatocytes limits liver
cancer development. Nature.

[R48] Klaffl S, Eikmanns BJ (2010). Genetic and functional analysis of the soluble oxaloacetate
decarboxylase from Corynebacterium glutamicum. J Bacteriol.

[R49] Kuchay M (2016). Hypercalcemia of advanced chronic liver disease: a forgotten
clinical entity!. Clin Cases Miner Bone Metab.

[R50] Kuro-o M, Matsumura Y, Aizawa H, Kawaguchi H, Suga T, Utsugi T (1997). Mutation of the mouse klotho gene leads to a syndrome resembling
ageing. Nature.

[R51] Laporta J, Peters TL, Weaver SR, Merriman KE, Hernandez LL (2013). Feeding 5-hydroxy-l-tryptophan during the transition from
pregnancy to lactation increases calcium mobilization from bone in
rats. Domest Anim Endocrinol.

[R52] Lesnik C, Golani-Armon A, Arava Y (2015). Localized translation near the mitochondrial outer membrane: an
update. RNA Biol.

[R53] Lietzan AD, St Maurice M (2014). Functionally diverse biotin-dependent enzymes with oxaloacetate
decarboxylase activity. Arch Biochem Biophys.

[R54] Manjasetty BA, Niesen FH, Delbrück H, Götz F, Sievert V, Büssow K (2004). X-ray structure of fumarylacetoacetate hydrolase family member
Homo sapiens FLJ36880. Biol Chem.

[R55] Martin N, Bernard D (2018). Calcium signaling and cellular senescence. Cell Calcium.

[R56] Minisola S, Pepe J, Piemonte S, Cipriani C (2015). The diagnosis and management of hypercalcaemia. BMJ (Clin Res Ed).

[R57] Nakayama S, Kretsinger RH (1994). Evolution of the EF-hand family of proteins. Annu Rev Biophys Biomol Struct.

[R58] Oka S, Shiraishi Y, Yoshida T, Ohkubo T, Sugiura Y, Kobayashi Y (2004). NMR structure of transcription factor Sp1 DNA binding
domain. Biochemistry.

[R59] Oudshoorn C, van der Cammen TJM, McMurdo MET, van Leeuwen JPTM, Colin EM (2009). Ageing and vitamin D deficiency: effects on calcium homeostasis
and considerations for vitamin D supplementation. Br J Nutr.

[R60] Parys JB, Bultynck G (2018). Ca2+ signaling and cell death: focus on the role of Ca2+ signals
in the regulation of cell death & survival processes in health,
disease and therapy. Cell Calcium.

[R61] Patergnani S, Suski JM, Agnoletto C, Bononi A, Bonora M, De Marchi E (2011). Calcium signaling around mitochondria associated membranes
(MAMs). Cell Commun Signal.

[R62] Pelley JW (2007). Glycolysis and pyruvate oxidation. Elsevier’s Integrated
Biochemistry. Elsevier.

[R63] Perocchi F, Gohil VM, Girgis HS, Bao XR, McCombs JE, Palmer AE (2010). MICU1 encodes a mitochondrial EF hand protein required for Ca2+
uptake. Nature.

[R64] Petit M, Koziel R, Etemad S, Pircher H, Jansen-Dürr P (2017). Depletion of oxaloacetate decarboxylase FAHD1 inhibits
mitochondrial electron transport and induces cellular senescence in human
endothelial cells. Exp Gerontol.

[R65] Pircher H, Straganz GD, Ehehalt D, Morrow G, Tanguay RM, Jansen-Dürr P (2011). Identification of human Fumarylacetoacetate Hydrolase
Domain-containing Protein 1 (FAHD1) as a novel mitochondrial
acylpyruvase. J Biol Chem.

[R66] Pircher H, von Grafenstein S, Diener T, Metzger C, Albertini E, Taferner A (2015). Identification of FAH domain-containing protein 1 (FAHD1) as
oxaloacetate decarboxylase. J Biol Chem.

[R67] Pitts MW, Hoffmann PR (2018). Endoplasmic reticulum-resident selenoproteins as regulators of
calcium signaling and homeostasis. Cell Calcium.

[R68] Ran T, Wang Y, Xu D, Wang W (2011). Expression, purification, crystallization and preliminary
crystallographic analysis of Cg1458: a novel oxaloacetate decarboxylase from
Corynebacterium glutamicum. Acta Crystallogr Sect F Struct Biol Cryst Commun.

[R69] Ran T, Gao Y, Marsh M, Zhu W, Wang M, Mao X (2013). Crystal structures of Cg1458 reveal a catalytic lid domain and a
common catalytic mechanism for the FAH family. Biochem J.

[R70] Schinkmann K, Li C (1992). Localization of FMRFamide-like peptides in Caenorhabditis
elegans. J Comp Neurol.

[R71] Seki N, Moczko M, Nagase T, Zufall N, Ehmann B, Dietmeier K (1995). A human homolog of the mitochondrial protein import receptor
Mom19 can assemble with the yeast mitochondrial receptor
complex. FEBS Lett.

[R72] Shyn SI, Kerr R, Schafer WR (2003). Serotonin and go modulate functional states of neurons and
muscles controlling C. Elegans egg-laying behavior Curr Biol.

[R73] Starkov AA (2010). The molecular identity of the mitochondrial Ca2+ sequestration
system. FEBS J.

[R74] Stöckl P, Hütter E, Zwerschke W, Jansen-Dürr P (2006). Sustained inhibition of oxidative phosphorylation impairs cell
proliferation and induces premature senescence in human
fibroblasts. Exp Gerontol.

[R75] Taferner A, Pircher H, Koziel R, von Grafenstein S, Baraldo G, Palikaras K (2015). FAH domain containing protein 1 (FAHD-1) is required for
mitochondrial function and locomotion activity in C. elegans. PLoS One.

[R76] Takeuchi A, Kim B, Matsuoka S (2015). The destiny of Ca2+ released by mitochondria. J Physiol Sci.

[R77] Timm DE, Mueller HA, Bhanumoorthy P, Harp JM, Bunick GJ (1999). Crystal structure and mechanism of a carbon-carbon bond
hydrolase. Structure (London, England: 1993).

[R78] Trent C, Tsuing N, Horvitz HR (1983). Egg-laying defective mutants of the nematode Caenorhabditis
elegans. Genetics.

[R79] Uhlen M, Björling E, Agaton C, Szigyarto CA-K, Amini B, Andersen E (2005). A human protein atlas for normal and cancer tissues based on
antibody proteomics. Mol Cell Proteom.

[R80] Uhlen M, Oksvold P, Fagerberg L, Lundberg E, Jonasson K, Forsberg M (2010). Towards a knowledge-based human protein atlas. Nat Biotechnol.

[R81] Uhlen M, Fagerberg L, Hallstrom BM, Lindskog C, Oksvold P, Mardinoglu A (2015). Tissue-based map of the human proteome. Science.

[R82] Urakawa I, Yamazaki Y, Shimada T, Iijima K, Hasegawa H, Okawa K (2006). Klotho converts canonical FGF receptor into a specific receptor
for FGF23. Nature.

[R83] van der Wulp MYM, Verkade HJ, Groen AK (2013). Regulation of cholesterol homeostasis. Mol Cell Endocrinol.

[R84] Veldurthy V, Wei R, Oz L, Dhawan P, Jeon YH, Christakos S (2016). Vitamin D, calcium homeostasis and aging. Bone Res.

[R85] Walther DM, Rapaport D (2009). Biogenesis of mitochondrial outer membrane
proteins. Biochim Biophys Acta (BBA) – Mol Cell Res.

[R86] Wang W-A, Liu W-X, Durnaoglu S, Lee S-K, Lian J, Lehner R (2017). Loss of calreticulin uncovers a critical role for calcium in
regulating cellular lipid homeostasis. Sci Rep.

[R87] Wasmuth EV, Lima CD (2017). UniProt: the universal protein knowledgebase. Nucleic Acids Res.

[R88] Waterhouse A, Bertoni M, Bienert S, Studer G, Tauriello G, Gumienny R (2018). SWISS-MODEL: homology modelling of protein structures and
complexes. Nucleic Acids Res.

[R89] Weaver SR, Prichard AP, Endres EL, Newhouse SA, Peters TL, Crump PM (2016). Elevation of circulating serotonin improves calcium dynamics in
the peripartum dairy cow. J Endocrinol.

[R90] Weinshenker D, Garriga G, Thomas JH (1995). Genetic and pharmacological analysis of neurotransmitters
controlling egg laying in C. elegans. J Neurosci.

[R91] Weiss AKH, Naschberger A, Loeﬄer JR, Gstach H, Bowler MW, Holzknecht M (2018a). Structural basis for the bi-functionality of human oxaloacetate
decarboxylase FAHD1. Biochem J.

[R92] Weiss AKH, Loeﬄer JR, Liedl KR, Gstach H, Jansen-Dürr P (2018b). The fumarylacetoacetate hydrolase (FAH) superfamily of enzymes:
multifunctional enzymes from microbes to mitochondria. Biochem Soc Trans.

[R93] Weiss AKH, Holzknecht M, Cappuccio E, Dorigatti I, Kreidl K, Naschberger A (2019). Expression, purification, crystallization, and enzyme assays of
fumarylacetoacetate hydrolase domain-containing proteins. J Vis Exp.

[R94] Weiss AKH, Naschberger A, Cappuccio E, Metzger C, Mottes L, Holzknecht M (2020). Structural and functional comparison of fumarylacetoacetate
domain containing protein 1 in human and mouse. Biosci Rep.

[R95] Wiel C, Lallet-Daher H, Gitenay D, Gras B, Le Calvé B, Augert A (2014). Endoplasmic reticulum calcium release through ITPR2 channels
leads to mitochondrial calcium accumulation and senescence. Nat Commun.

[R96] Wiley CD, Velarde MC, Lecot P, Liu S, Sarnoski EA, Freund A (2016). Mitochondrial dysfunction induces senescence with a distinct
secretory phenotype. Cell Metab.

[R97] Zheng W, Wuyun Q, Li Y, Mortuza SM, Zhang C, Pearce R (2019). Detecting distant-homology protein structures by aligning deep
neural-network based contact maps. PLoS Comput Biol.

