# Supplementary material for: Regulation of cellular senescence by eukaryotic members of the FAH superfamily – A role in calcium homeostasis?
Source: Mech Ageing Dev. Author manuscript; Available in PMC 2020 Dec 14. (PMC7116474; doi:10.1016/j.mad.2020.111284)
Supplement: suppl. files SF1-SF3, suppl. figures S1-S3 [file EMS107273-supplement-suppl__files_SF1_SF3__suppl__figures_S1_S3.zip › 1-s2.0-S0047637420300804-mmc1.docx]

**SUPPLEMENTARY MATERIAL**

**Regulation of cellular senescence by eukaryotic members of the FAH superfamily – a role in calcium homeostasis?**

*Alexander K. H. Weiss^1,2,*^, Eva Albertini^1,2^, Max Holzknecht^1,2^, Elia Cappuccio^1,2^,
Ilaria Dorigatti^1,2^, Anna Krahbichler^1,2^, Elisabeth Damisch^1,2^,
Hubert Gstach^3^, Pidder Jansen-Dürr^1,2^*

1. University of Innsbruck; Research Institute for Biomedical Aging Research;
   Rennweg 10, A-6020 Innsbruck, Austria
2. University of Innsbruck; Center for Molecular Biosciences Innsbruck (CMBI), Austria
3. University of Vienna; UZ2 E349, Department of Pharmaceutical Chemistry, Faculty of Life Sciences, Althanstrasse 14, 1090 Vienna, Austria

*) corresponding author

**Materials and Methods**

[Prediction of protein properties 3](#_Toc40205788)

[Motif search and comparison 3](#_Toc40205789)

[Supplementary files SF1: 3](#_Toc40205790)

[Supplementary files SF2: 3](#_Toc40205791)

[Supplementary files SF3: 4](#_Toc40205792)

[Figure S1: 5](#_Toc40205793)

[Figure S2: 6](#_Toc40205794)

[Figure S3: 7](#_Toc40205795)

[References: 8](#_Toc40205796)

# Prediction of protein properties

Protein properties such as stability and complex formation were predicted using the *ProtParam*^1^ server and the *UniProt*^2^ database. Ion ligand binding prediction was performed using the *IonCom*^3,4^ server, by aligning deep neural-network based contact maps created from the 3D data of a PDB model. The supplementary material contains information about mitochondrial enzymes listed in the *MitoCarta2*^5^ database, that were used for a general stability analysis automated with *BioPython*^6^. Figure S1 and S2 summarize this data.

# Motif search and comparison

The supplementary material contains information about selected sequences of known EF-hand domain-containing proteins including all isoforms that were used for *BLASTp* alignment with FAHD1. The *UniProt*^2^ database lists curated entries of human proteins with mitochondrial signal-anchor motifs (keyword Signal-anchor KW-0735). Protein motifs were investigated *via* *TargetP-2.0*^7^ server that predicts the presence of conserved mitochondrial transit peptide sequences. Homology modelling of the protein structure of FAHD2a was performed *via* *Swiss-Model*^8^ homology modelling.

# Supplementary files SF1:

*MitoCarta2 data.xlsx; MitoCarta2 data.fasta*

An Excel file containing information about mitochondrial enzymes listed in the *MitoCarta2*^5^ database, that was used to compute protein properties using the *ProtParam*^1^ server and the *UniProt*^2^ database. Associated FASTA data is provided in a second file. Figure S1 displays the *BioPython*^6^ script used to gather this information. Figure S2 displays the final data obtained.

# Supplementary files SF2:

*EF hand proteins.fasta*

Sequences of known EF-hand domain-containing proteins including all isoforms (ACTN1, ACTN2, ACTN3, ACTN4, APBA2BP, AYTL1, AYTL2, C14orf143, CABP1, CABP2, CABP3, CABP4, CABP5, CABP7, CALB1, CALB2, CALM2, CALM3, CALML3, CALML4, CALML5, CALML6, CALN1, CALU, CAPN1, CAPN11, CAPN2, CAPN3, CAPN9, CAPNS1, CAPNS2, CAPS, CAPS2, CAPSL, CBARA1, CETN1, CETN2, CETN3, CHP, CHP2, CIB1, CIB2, CIB3, CIB4, CRNN, DGKA, DGKB, DGKG, DST, DUOX1, DUOX2, EFCAB1, EFCAB2, EFCAB4A, EFCAB4B, EFCAB6, EFCBP1, EFCBP2, EFHA1, EFHA2, EFHB, EFHC1, EFHD1, EFHD2, EPS15, EPS15L1, FKBP10, FKBP14, FKBP7, FKBP9, FKBP9L, FREQ, FSTL1, FSTL5, GCA, GPD2, GUCA1A, GUCA1B, GUCA1C, HPCAL1, HPCAL4, HZGJ, IFPS, ITSN1, ITSN2, KCNIP1, KCNIP2, KCNIP3, KCNIP4, KIAA1799, LCP1, MACF1, MRLC2, MRLC3, MST133, MYL1, MYL2, MYL5, MYL6B, MYL7, MYL9, MYLC2PL, MYLPF, NCALD, NIN, NKD1, NKD2, NLP, NOX5, NUCB1, NUCB2, OCM, PDCD6, PEF1, PKD2, PLCD1, PLCD4, PLCH1, PLCH2, PLS1, PLS3, PP1187, PPEF1, PPEF2, PPP3R1, PPP3R2, PRKCSH, PVALB, RAB11FIP3, RASEF, RASGRP, RASGRP1, RASGRP2, RASGRP3, RCN1, RCN2, RCN3, RCV1, RCVRN, REPS1, RHBDL3, RHOT1, RHOT2, RPTN, RYR2, RYR3, S100A1, S100A11, S100A12, S100A6, S100A8, S100A9, S100B, S100G, S100Z, SCAMC-2, SCGN, SCN5A, SDF4, SLC25A12, SLC25A13, SLC25A23, SLC25A24, SLC25A25, SPATA21, SPTA1, SPTAN1, SRI, TBC1D9, TBC1D9B, TCHH, TESC, TNNC1, TNNC2, USP32, VSNL1, ZZEF1). This data was used to perform a *BLASTp* analysis against human FAHD1 (see Figure 3).

# Supplementary files SF3:

*signal_anchor.fasta; signal_anchor - alignment.txt; TargetP FAHD2a.txt*

The *UniProt*^2^ database lists curated (reviewed) entries of human proteins with signal-anchor motifs (keyword Signal-anchor KW-0735). *BLASTp* analysis of human FAHD1 and proteins in this selection displays sequence similarities of 28 % to 50 % with 8 entries, mapping to 3 proteins and their isoforms. Alignment displays sequence identity in the amino acid ranges 1-24, 26-84, 27-131, and 185-207 of human FAHD1. This data may suggest a mechanism by which FAHD1 is synthetized in the cytosol and incorporated into mitochondria as a signal-anchored protein.

# Figure S1:


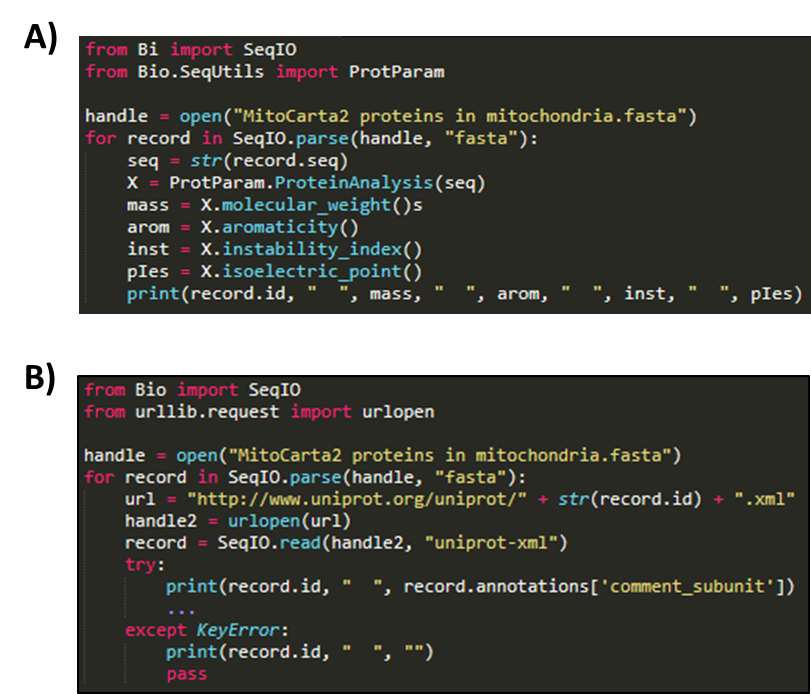


**Figure S1: BiosPython scripts used to gather information about mitochondrial proteins.**

Source code (*BioPython*^6^) that was used **A)** to gather information from the *ProtParam*^1^ server, and **B)** to gather information from *UniProt*^2^. Proteins were selected by reported mitochondrial localization from the *MitoCarta2*^5^ database.

# Figure S2:


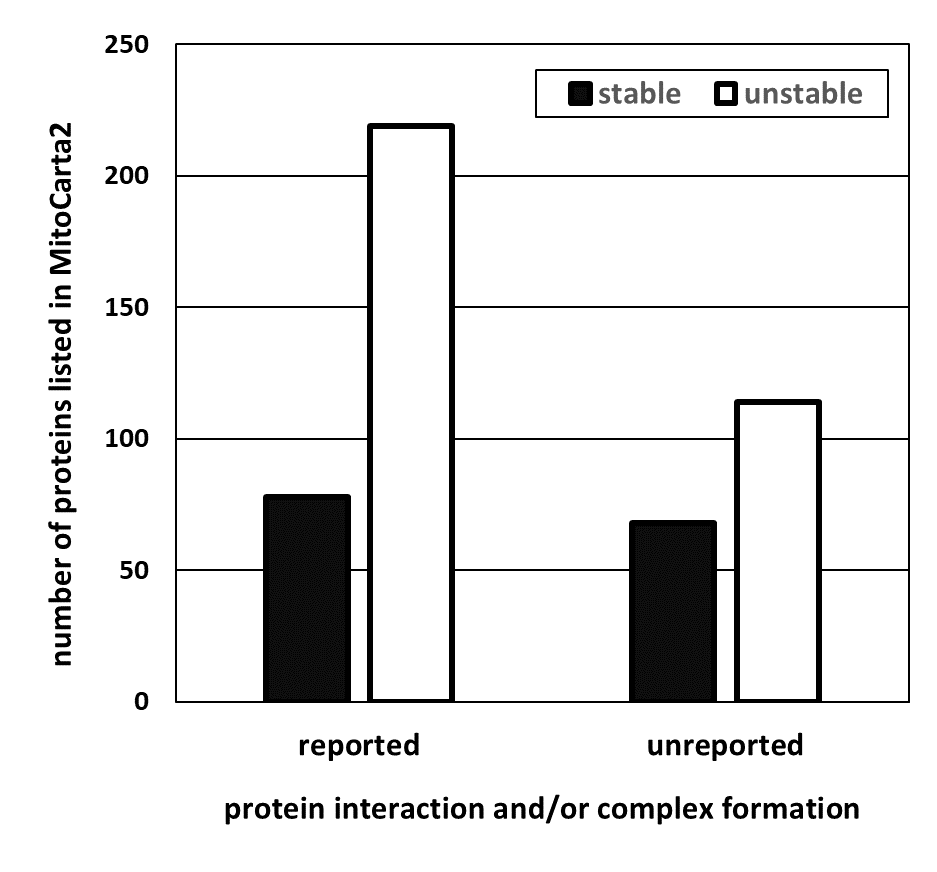


**Figure S2: Stability of mitochondrial proteins, sorted by reported protein interaction**

Mitochondrial proteins listed in the *MitoCarta2*^5^ database were classified using the *ProtParam*^1^ server and *UniProt*^2^ database. Information about predicted protein stability is compared to reported protein interaction and/or cluster formation: Displayed is the number of proteins that are either reported or unreported to interact with other proteins and/or are part of protein complexes, each group differentiated by the predicted stability. The number of predicted stable proteins is similar in both groups. The number of predicted unstable proteins that are reported to interact with other proteins is about twice the number of unstable proteins that are not reported to cluster. We may conclude that there is a trend of unstable proteins in solution to form protein complexes and/or to interact with other proteins. Although the protein model (PDB data) of FAHD1 describes a homodimer, we may conclude from this data that an involvement of FAHD1 in protein interactions is rather plausible.

# Figure S3:


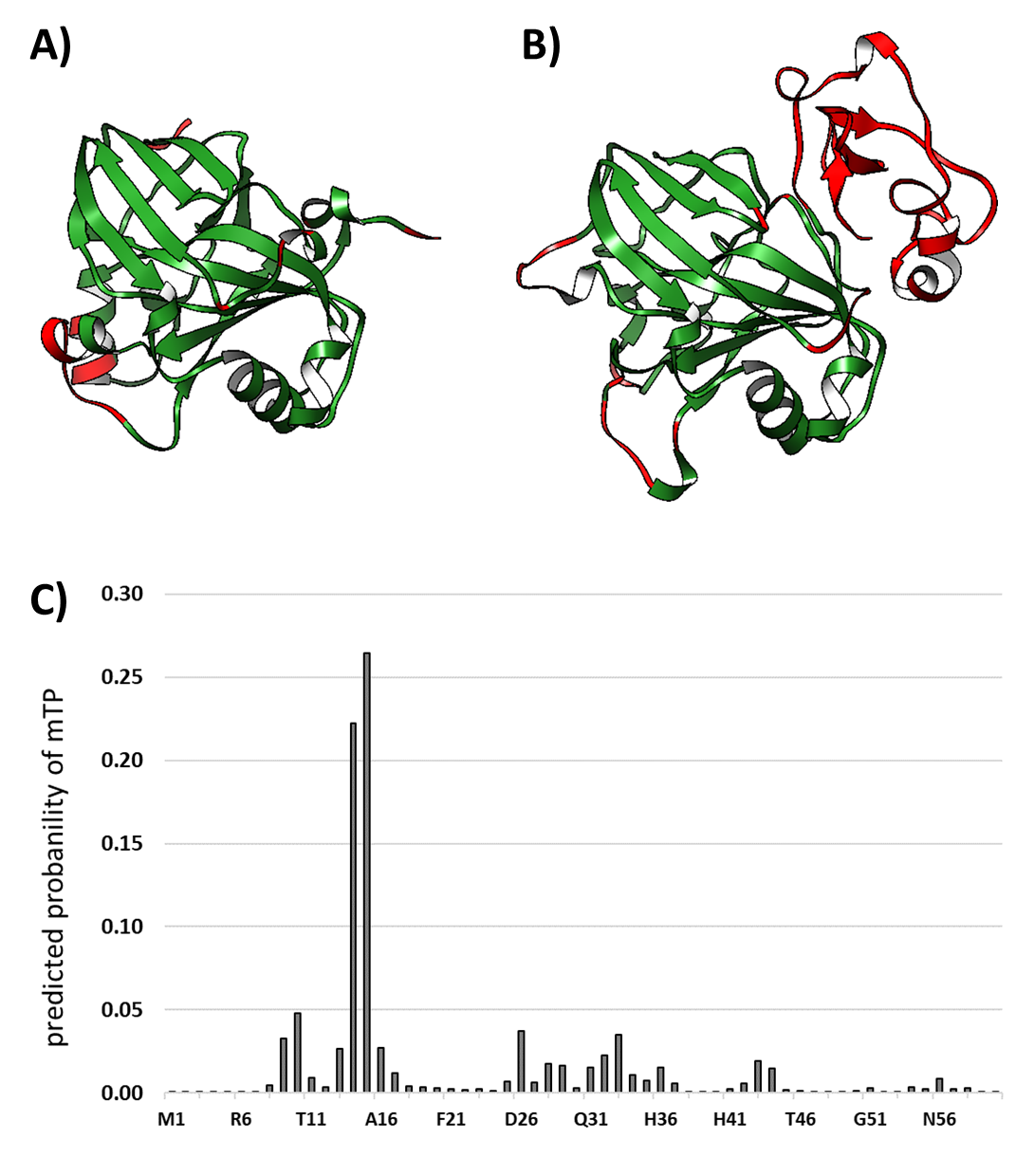


**Figure S3: Homology modelling of the protein structure of FAHD2a.**

The resolved structure of human FAHD1^9^ (**panel A**) was used as template for a *Swiss-Model*^8^ homology modelling of the protein structure of FAHD2a (**panel B**), i.e., this virtual model is not a crystal structure. Apparent differences in the tertiary structure are denoted by red color. The major two differences are a slightly different folding in the anyways very flexible loop region around R32 (lower left in both panels), and the *N*-terminal hydrophobic region of FAHD2a (upper right in the right panel). All structure motifs that have been identified to be of importance for the catalytic activity of FAHD1 are fully conserved. The *TargetP-2.0*^7^ server predicts the presence of a conserved mitochondrial transit peptide sequence (mTP) (**panel C**) around L14 of FAHD2a and FAHD2b, but not in the sequence of FAHD1.

# References:

1. Gasteiger E., Hoogland C., Gattiker A., Duvaud S., Wilkins M.R., Appel R.D., B. A. Protein Identification and Analysis Tools on the ExPASy Server. in *The Proteomics Protocols Handbook* (ed. John M. Walker) 571–607 (Humana Press, 2005).

2. Wasmuth, E. V & Lima, C. D. UniProt: the universal protein knowledgebase. *Nucleic Acids Research* **45,** D158–D169 (2017).

3. Zheng, W., Wuyun, Q., Li, Y., Mortuza, S. M., Zhang, C., Pearce, R., *et al.* Detecting distant-homology protein structures by aligning deep neural-network based contact maps. *PLOS Computational Biology* **15,** e1007411 (2019).

4. Hu, X., Dong, Q., Yang, J. & Zhang, Y. Recognizing metal and acid radical ion-binding sites by integrating ab initio modeling with template-based transferals. *Bioinformatics (Oxford, England)* **32,** 3260–3269 (2016).

5. Calvo, S. E., Clauser, K. R. & Mootha, V. K. MitoCarta2.0: an updated inventory of mammalian mitochondrial proteins. *Nucleic Acids Research* **44,** D1251–D1257 (2016).

6. Cock, P. J. A., Antao, T., Chang, J. T., Chapman, B. A., Cox, C. J., Dalke, A., *et al.* Biopython: freely available Python tools for computational molecular biology and bioinformatics. *Bioinformatics* **25,** 1422–1423 (2009).

7. Almagro Armenteros, J. J., Salvatore, M., Emanuelsson, O., Winther, O., von Heijne, G., Elofsson, A., *et al.* Detecting sequence signals in targeting peptides using deep learning. *Life Science Alliance* **2,** e201900429 (2019).

8. Waterhouse, A., Bertoni, M., Bienert, S., Studer, G., Tauriello, G., Gumienny, R., *et al.* SWISS-MODEL: homology modelling of protein structures and complexes. *Nucleic Acids Research* **46,** W296–W303 (2018).

9. Weiss, A. K. H., Naschberger, A., Loeffler, J. R., Gstach, H., Bowler, M. W., Holzknecht, M., *et al.* Structural basis for the bi-functionality of human oxaloacetate decarboxylase FAHD1. *Biochemical Journal* **475,** 3561–3576 (2018).
